# Modeling medulloblastoma pathogenesis and treatment in human cerebellar organoids

**DOI:** 10.1101/gad.353292.125

**Published:** 2026-06-01

**Authors:** Thomas Willott, James G. Nicholson, Xinyu Zhang, Yunchen Xiao, Olumide Ogunbiyi, Benjamin Draper, Talisa Mistry, Laura K. Donovan, Ashirwad Merve, Nicolae Radu Zabet, Sara Badodi, Silvia Marino

**Affiliations:** 1 Brain Tumour Research Centre, Blizard Institute, Queen Mary University of London, London E1 2AT, United Kingdom;; 2 Department of Histopathology, Great Ormond Street Hospital for Children National Health Services Foundation Trust, London WC1N 3JH, United Kingdom;; 3 University College London Great Ormond Street Institute of Child Health, London WC1N 1EH, United Kingdom;; 4 Barts Brain Tumour Centre, Queen Mary University of London and Barts Health, London E1 2AT, United Kingdom

**Keywords:** cerebellar development, cell of origin, cerebellar organoids, medulloblastoma

## Abstract

Medulloblastoma (MB) groups 3 and 4 arise from human-specific developmental contexts that remain experimentally inaccessible, limiting mechanistic insight into tumor initiation. We generated cerebellar organoids (CbOs) from expanded potential stem cells (EPSCs) and performed integrated single-cell transcriptomic and DNA methylation analyses, revealing discrete human rhombic lip progenitor populations whose developmental programs align with group 3- and group 4-associated lineages. Using c-*MYC* overexpression as an initiating oncogenic event, we demonstrated that these lineage-restricted progenitors are susceptible to neoplastic transformation, generating tumors with group 3 MB molecular features in vitro and in vivo. We further established a CbO-MB coculture system that preserves malignant and nonmalignant cellular compartments, hence enabling interrogation of tumor–microenvironment interactions and providing simultaneous readouts of antitumor efficacy and cerebellar toxicity upon drug treatment. Receptor–ligand modeling in this system identifies TGFβ paracrine signaling as a microenvironmental pathway supporting MB growth, which we validated pharmacologically. Further analyses of cocultured MB cells revealed a myogenic differentiation program enriched in *MYC*-driven MB and recurrent disease, which is associated with poorer prognosis. Together, this work establishes a collection of CbO models for studying group 3/4 MB initiation, growth, and microenvironmental dependencies and provides a tractable platform for further mechanistic and therapeutic investigation of these tumors.

## Abstract (precis)

In this study, Willott et al. use cerebellar organoids to recapitulate the development of the human cerebellum and model group 3/4 medulloblastoma (MB). This cerebellar organoid system harbored MB cells of origin capable of undergoing neoplastic transformation as well as sustained MB progression, providing a valuable preclinical tool to assess tumor–microenvironment interactions and therapeutic response.

Medulloblastoma (MB) is a cerebellar tumor accounting for the largest proportion of brain cancer diagnoses in children. Molecular subgrouping based on transcriptomic, epigenomic, and genomic features has collectively identified four MB subgroups—sonic hedgehog (SHH), Wingless-related integration site (WNT), group 3 (G3), and group 4 (G4)—each further divided into subtypes with distinct prognoses and responses to therapy (Cavalli et al. 2017; Northcott et al. 2017; Schwalbe et al. 2017; Sharma et al. 2019; Moss et al. 2023). Despite these advances in classification, MB standard of care remains unchanged, with patients undergoing surgical resection followed by chemotherapy and/or radiotherapy (Mueller and Chang 2009), which, albeit effective in a majority of patients, is associated with significant side effects.

The origins and molecular alterations for SHH and WNT MB are well characterized, enabling the development of molecularly targeted therapies and/or immunotherapies in preclinical models, which are now beginning to translate into early-phase clinical trials (Rechberger et al. 2023). In contrast, the unclear origins and lack of universal pathway aberrations or faithful preclinical models in G3/4 MB subgroups have hampered the identification of new treatment options (Thompson et al. 2020). The maintenance of primary MB cells as patient-derived orthotopic xenograft (PDX) models has overcome some of these issues, particularly for G3 MB (Shu et al. 2008; Sandén et al. 2017; Brabetz et al. 2018); however, the cost and resources required for these models limit their broader applications and scalability.

MB formation is closely linked to deregulated cerebellar development (Marino 2005). Recent studies identified spatiotemporally restricted mitotic glutamatergic neuron progenitors ∼12–14 postconception weeks (PCW) in the rhombic lip subventricular zone (RL^SVZ^) harboring photoreceptor cell (PRC) and unipolar brush cell (UBC) gene signatures as putative G3 and G4 MB cells of origin, respectively (Hendrikse et al. 2022; Smith et al. 2022). These progenitors are halted in development prior to cell cycle exit, priming them to malignant transformation. Importantly, immunohistochemical and pseudotime comparisons of relevant stages of human and murine cerebellar development showed significantly fewer of these proliferative progenitors in mice, raising the possibility that mouse models may not allow the study of key features of G3/4 MB, including their initiation (Hendrikse et al. 2022; Smith et al. 2022). More recently, neural stem cells (NSCs) in the RL^VZ^ harboring a *PRTG*^+^*;SOX2*^+^*;MYC*^high^*;NESTIN*^low^ signature have been linked to G3 MB onset (Visvanathan et al. 2024). These studies suggest that the failed maturation of early cerebellar cell lineages at a specific subcompartment location and developmental stage is linked to the ontogeny of G3/4 MB tumors. In keeping with these findings, single-cell multiomics profiling and genetic modeling estimated the onset of G3/4 MB tumors in the first gestational trimester (Okonechnikov et al. 2025), though suitable experimental models to validate and further dissect tumor initiation and progression pertinent to the formation of these MB subgroups have so far been lacking.

Advances in organoid research have enabled the generation of new tools to study various aspects of cancer biology, including tumor initiation (Bian et al. 2018) and metastasis (Choe et al. 2020) and as a coculture model for drug testing (Linkous et al. 2019), with important implications for personalized medicine (Vinel et al. 2021). Methods to generate cerebellar organoids (CbOs) from induced pluripotent stem cells (iPSCs) have been developed (Muguruma et al. 2015; Silva et al. 2020) and characterized at the single-cell transcriptome level, achieving neuronal and glial differentiation with cerebellar subcompartment identity (Nayler et al. 2021; Chen et al. 2023; Atamian et al. 2024). To date, CbO-based models of MB have encompassed bulk-engineered organoids without cell of origin specificity (Ballabio et al. 2020; van Essen et al. 2024) or coculture systems with established SHH MB cell lines (van Essen et al. 2025).

Here, we have generated human CbOs from expanded potential stem cells (EPSCs) and assessed their suitability to model G3/4 MB. We have shown that they contain the putative MB cells of origin at stages of differentiation comparable with fetal cerebellar development and demonstrated that these cells can be genetically engineered to induce neoplastic transformation. In addition, we have analyzed the suitability of CbOs as a coculture model of G3/4 MB and demonstrated their value as a preclinical drug testing tool while providing further insight into MB transcriptional state heterogeneity.

## Results

### CbOs recapitulate the epigenetic development of the human cerebellum

**Figure 1.**
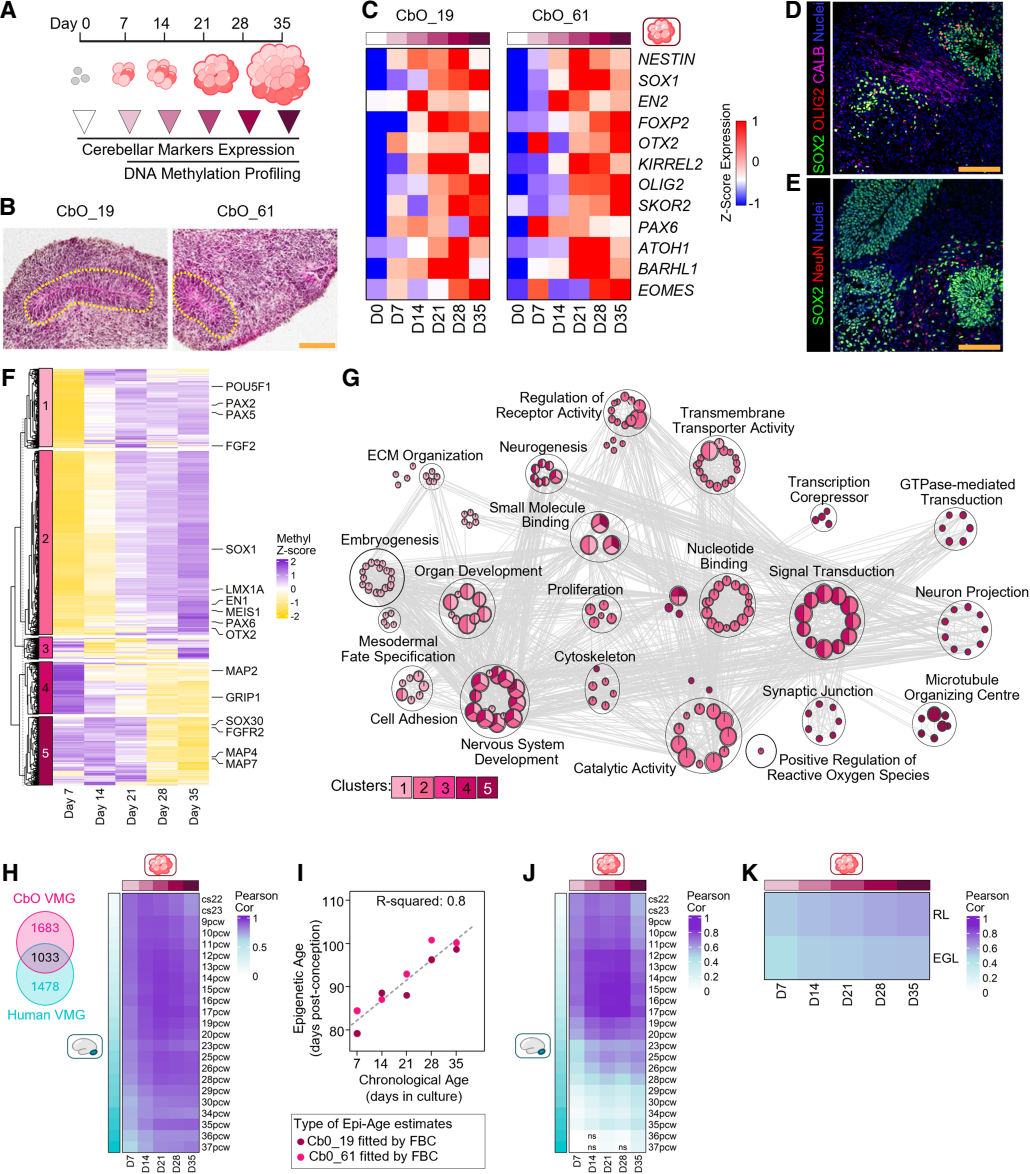
CbOs recapitulate human cerebellum epigenetic development. (*A*) Schematic of CbO time points collected to assess cerebellar marker expression and DNA methylation profiles. (*B*) H&E staining of CbO_19 and CbO_61, with flat oval neural rosettes circled. Scale bar, 150 µm. (*C*) Heat maps showing average expression of marker genes associated with cerebellar differentiation up to day 35 (D35) of development for CbO_19 and CbO_61 derived from two independent EPSC lines (D0). Heat maps show *Z*-scores for average ΔCT values for three CbOs taken from *n* = 3 independent batches (see Supplemental Fig. S1C). (*D*) Representative immunofluorescence staining of OLIG2^+^ GABAergic progenitors (red), Calbindin^+^ (CALB^+^) mature neurons (pink), and SOX2^+^ neural stem and progenitor cells (green) in D35 CbO_61. Scale bar, 100 µm. (*E*) Representative immunofluorescence staining of NeuN^+^ glutamatergic neuronal cells (red) and SOX2^+^ neural stem and progenitor cells (green) in D35 CbO_61. Scale bar, 100 µm. (*F*) Heat map showing *k*-means clustering (*k* = 5) of significant variable methylated genes across 35 days of CbO maturation. Mean probe β-values were calculated for each time point from pooled samples (*n* = 3) of CbO_19 and CbO_61. *Z*-scores are plotted for average β-values of probes contained within each gene. (*G*) Bubble plot showing the top 100 ranked significant GO biological processes and molecular functions of genes from five clusters shown in *F* (adjusted *P* < 0.05). Bubbles are colored by cluster, and size is proportional to the number of genes in the GO term. (*H*, *left*) Venn diagram showing 1033 common variably methylated genes (VMGs) calculated through human (Carnegie stage 22–37 postconception weeks [PCW]) and CbO (CbO_19 and CbO_61, days 7–35) development. (*Right*) Heat map showing Pearson correlation of human and CbO samples on their β-values for 1033 common VMGs. All Pearson correlations are *P* < 0.001. (*I*) Regression plot between the chronological ages of CbO_19 and CbO_61 and modeled epigenetic ages from the fetal brain clock (Steg et al. 2021). (*J*) Heat map showing Pearson correlation of the β-value of the top 1% (6886) age-dependent CpG probes between human developing cerebellum samples and CbOs. All Pearson correlations are *P* < 0.001 unless nonsignificant (ns) is indicated. (*K*) Heat map showing Pearson correlation of average β-values of CbOs to human microdissected rhombic lip (RL) and external granule layer (EGL) regions (Hendrikse et al. 2022) for differentially methylated probes calculated between RL and EGL samples (*n* = 6 and *n* = 7, respectively). All Pearson correlations are *P* < 0.001.

CbOs were generated from two previously characterized EPSC lines (CbO19 and CbO61) that were reprogrammed from dura mater fibroblasts (Yang et al. 2017; Vinel et al. 2021). Organoid differentiation was followed for 35 days, according to previously published iPSC-derived protocols (Fig. 1A; Supplemental Fig. S1A; Muguruma et al. 2015; Silva et al. 2020), demonstrating sustained growth (Supplemental Fig. S1B) and forming characteristic flat oval neural rosettes by day 35 (D35) of culture (Fig. 1B). By D7, CbOs had lost expression of the EPSC marker *NANOG*, and from D7–D14, they expressed NSC markers *NESTIN* and *SOX1* as well as the cerebellar homeobox and developmental genes *EN2*, *FOXP2*, and *OTX2* (Frantz et al. 1994). *KIRREL2* and *PAX6*, markers of early Purkinje or granule cell progenitors, were expressed at D7–D14, followed by markers of more mature precursors, including *OLIG2/SKOR2* and *ATOH1/BARHL1* at D21–D28, indicating the presence of GABAergic and glutamatergic lineages, respectively. The transcription factor *EOMES*, expressed in glutamatergic progenitors and later in UBCs, peaked in later stages (D28–D35) of CbO maturation (Fig. 1C; Supplemental Fig. S1C). The presence of GABAergic and glutamatergic granule neurons was confirmed by expression of CALBINDIN and NeuN proteins, respectively, proximal to SOX2-positive neural rosettes at D35 (Fig. 1D,E). Gene expression patterns were similar and consistent with reproducible differentiation into cerebellar cell lineages in CbOs derived from both EPSC lines (Fig. 1C; Supplemental Fig. S1C), which we used as biological replicates in this study.

DNA methylation regulates the spatiotemporal expression of cell lineage-specific genes during brain development (Badodi and Marino 2022), but methylation patterns in developing CbOs have yet to be explored. We therefore profiled DNA methylation of CbOs across D7–D35 of differentiation and maturation and identified genes showing significant variable methylation through CbO development (Fig. 1F). Grouping variably methylated genes (VMGs) by *k*-means clustering revealed time-dependent methylation programs, with clusters 1 and 2 containing transcription factors linked to early cerebellum development and lineage specification (*PAX2/5/6*, *LMX1A*, and *OTX2*) and accordingly displaying initial D7–D14 hypomethylation. In contrast, genes involved in neuronal differentiation and axon guidance (*MAP2/4/7* and *GRIP1)* were found in clusters 4 and 5 and became progressively hypomethylated during later CbO differentiation (Fig. 1F). GO pathway analysis of the identified VMGs revealed that early hypomethylated clusters were enriched for biological processes related to embryogenesis, fate specification, and organ development. Conversely, genes displaying later hypomethylation patterns were associated with neuronal differentiation, signal transduction, synaptic junctions, and neuron projections (Fig. 1G).

To benchmark CbO epigenetic development against the human cerebellum, we assembled a cohort of fetal cerebellar samples spanning all developmental stages (Carnegie stage 22–37 PCW) extending beyond the range of previous reference cohorts to identify epigenetic regulatory events throughout cerebellar development (Lister et al. 2013; Spiers et al. 2015). Analysis of human cerebellar samples (Supplemental Fig. S1D) revealed similar clustering of VMGs with distinct methylation patterns through human development (Supplemental Fig. S1E). GO analysis of these VMG clusters demonstrated shared earlier pathways with CbOs, including those involved in proliferation and tissue development, followed by later pathways associated with signal transduction, neuron projection, and catalytic activity (Supplemental Fig. S2F). Notably, we identified 1033 common VMGs between CbO and fetal cerebellar samples and observed broad, high correlation of their methylation status between humans and CbOs with some age-related variability (Fig. 1H), suggesting that the CbO epigenetic landscape is progressively developing in line with the human fetal cerebellum.

G3 and G4 MBs are thought to originate from human-specific RL progenitors first emerging in the developing cerebellum between 12 and 17 PCW (Hendrikse et al. 2022; Smith et al. 2022), and DNA methylation has been shown to correlate strongly with chronological age (Steg et al. 2021). Given the similarities between CbO and fetal cerebellum DNA methylation patterns, we next applied a fetal brain epigenetic clock algorithm to query whether our CbOs achieved sufficient maturation to coincide with the emergence of putative MB cells of origin (Steg et al. 2021). There was a strong positive correlation (*R*^2^ = 0.88, Pearson correlation *P* = 2 × 10^−5^) between CbO differentiation and their predicted epigenetic age, which suggests that by D35, CbOs achieve a level of maturation equivalent to 14 PCW (Fig. 1I). To corroborate this finding, we used our own extensive reference cohort of fetal cerebellar samples to identify the top 1% of all CpG probes (6886 probes) correlated with age and used the resulting age-associated epigenetic signature to compare cerebellar samples with CbOs (Fig. 1J). This analysis showed progressive maturation over time and found that by D35, CbOs showed high correlation to 12–17 PCW cerebellar samples. Finally, we compared our CbOs with methylation profiles from microdissected RL and EGL samples (Smith et al. 2022), revealing an increased correlation through CbO development and showing the highest correlation to RL with D35 CbOs (Fig. 1K).

Taken together, our data show that CbOs recapitulate human cerebellar epigenetic development and reach a level of maturation consistent with the emergence of G3/4 MB cells of origin by D35.

### CbOs harbor human-specific group 3 and group 4 medulloblastoma lineages of origin

**Figure 2.**
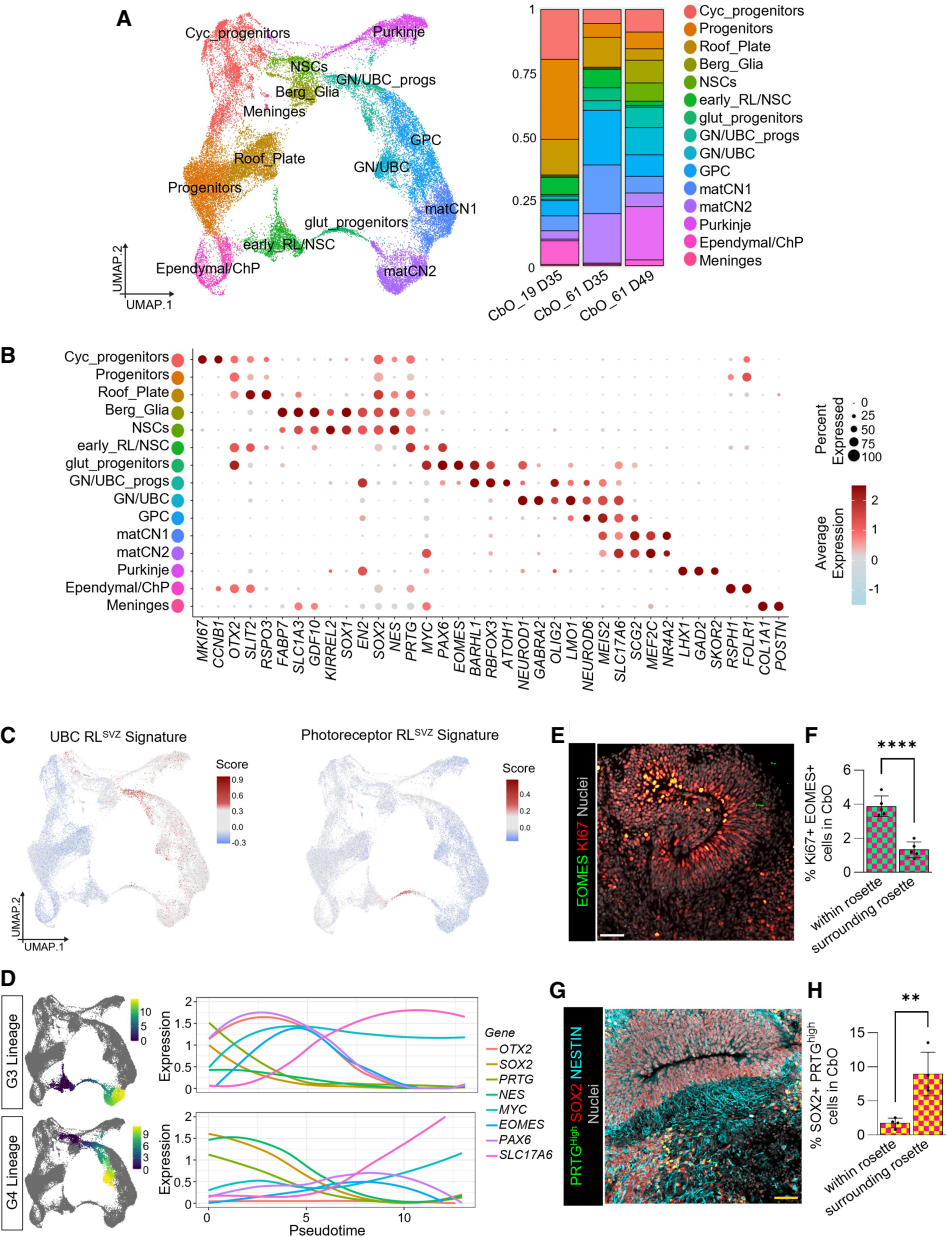
CbOs harbor human-specific group 3 and group 4 medulloblastoma cells of origin. (*A*) Uniform manifold approximation and projection (UMAP) plot of scRNA-seq data from CbO_19 and CbO_61 at day 35 and CbO_61 at day 49 of differentiation (pooled samples of *n* = 3 CbOs per time point) (*left*) and bar plots showing percentages of annotated cell populations identified (*right*). (*B*) Dot plot showing key marker genes used to identify major cell types in CbOs. (*C*) UMAP plot of scRNA-seq data in *A* showing signature score for unipolar brush cell (UBC) RL^SVZ^ signature genes (*left*) or photoreceptor RL^SVZ^ signature genes (*right*) (Smith et al. 2022). (*D*) Defined group 3 (G3) and 4 (G4) medulloblastoma cell of origin trajectories selected for further analysis (see *A*). (G3) Early_RL/NSC, glut_progs, and matCN2; (G4) NSCs, GN/UBC_progs, and GN/UBCs). Expression of genes associated with G3/4 medulloblastoma across each of the G3 and G4 trajectories plotted across pseudotime. (*E*) Representative immunohistochemistry of D35 CbO_61 showing a neural rosette with cells positive for EOMES (green) and Ki67 (red). Nuclei are shown in gray. Scale bar, 50 µm. (*F*) Quantification of staining shown in *E* of Ki67^+^EOMES^+^ nuclei with respect to CbO neural rosettes. Student's *t*-test: (****) *P* < 0.0001. *n* = 5 regions of interest quantified. (*G*) Representative immunohistochemistry of D35 CbO_61 showing a neural rosette with cells stained for PRTG (green), SOX2 (red), and NESTIN (light blue). Nuclei are shown in gray. Scale bar, 50 µm. (*H*) Quantification of staining shown in *G* of SOX2^+^PRTG^High^ nuclei with respect to CbO neural rosettes. PRTG^High^ is defined as the top 50% of the fluorescence signal. Student's *t*-test: (**) *P* < 0.01. *n* = 5 regions of interest quantified.

We next profiled D35 and D49 CbOs by scRNA-seq to determine their cellular composition at a time point consistent with the emergence of MB cells of origin (Fig. 2A). A substantial degree of cellular diversity was observed, and 17 clusters were identified (Supplemental Fig. S2A). Despite some differences in cell type proportions between samples, no clusters were specific to either CbO line or time point (Fig. 2A; Supplemental Fig. S2B), and samples were therefore analyzed together. Clusters were annotated based on their expression of marker genes (Fig. 2B) and correlations to endogenous cell types from reference data sets of human developing cerebellum (Supplemental Fig. S2C; Aldinger et al. 2021; Zhong et al. 2023; Sepp et al. 2024). We identified noncommitted *MKI67*^*+*^ and *CCN1B*^*+*^ cycling progenitors as well as *OTX2*^*+*^ and *SOX2*^*+*^ progenitor clusters. Two NSC clusters were identified: a broad cluster of *SOX1*^*+*^, *SOX2*^*+*^, *EN2*^*+*^, and *NES*^*+*^ NSCs and a more defined cluster expressing *SOX2*^*+*^, *PRTG*^*+*^, and *PAX6*^*+*^. We found several clusters associated with glutamatergic lineages, including two clusters of *BARHL1*^*+*^ and *RBFOX3*^*+*^ progenitors characterized by *PAX6* or *ATOH1* expression (glut_progenitors and GN/UBC_progs, respectively). Additionally, we detected a *NEUROD1*^*+*^ GN/UBC cluster also expressing mature glutamatergic neuronal markers (*LMO1* and *GABRA2*) and a *NEUROD6*^*+*^ and *MEIS2*^*+*^ glutamatergic progenitor cell (GPC) cluster. We further identified two mixed clusters of maturing cerebellar neurons (matCN1 and matCN2) positive for *MEIS2*, *SLC17A6*, and *NR4A2*, which are expressed by neurons located in the neural transitory zone (NTZ) during cerebellar development (Sepp et al. 2024). We further observed a neuronal cluster of GABAergic *SKOR2*^+^, *LHX1*^+^, and *GAD2*^+^ Purkinje cells as well as *FABP7*^*+*^ and *SLC1A3*^*+*^ Bergmann glial cells (Berg_Glia). In addition to cerebellar lineages, we also identified other hindbrain clusters containing cell types contributing to cerebellar development, including *FOLR1*^+^ and *RSPH1*^+^ ependymal/choroid plexus, *POST*^+^ and *COL1A1*^+^ meninges, and *SLIT2*^+^ and *RSPO3*^+^ roof plate cells (Fig. 2B). Comparison with published hiPSC-derived cerebellar organoids (Nayler et al. 2021) indicated that the two CbO culture approaches produced comparable cell types, with our system exhibiting a greater representation of mature neural populations (Fig. 2A; Supplemental Fig. S2D).

Putative G3/4 MB cells of origin were first described in the RL^SVZ^ as *EOMES*^*+*^ progenitors expressing either a photoreceptor-like (PRC.RL^SVZ^) or UBC-like (UBC.RL^SVZ^) expression signature, respectively (Hendrikse et al. 2022; Smith et al. 2022), with a more recent study reporting a *PRTG*^+^*;SOX2*^+^*;MYC*^high^*;NESTIN*^low^ stem cell population in the RL^VZ^ as alternative G3 cells of origin (Visvanathan et al. 2024). Consistent with our DNA methylation findings (Fig. 1K), we saw an enrichment of an RL^SVZ^ gene signature score in the glutamatergic cerebellar neuronal and progenitor clusters and RL^VZ^ gene signature enrichment in the less defined hindbrain/NSC clusters, suggesting that CbOs contain cells with cerebellar subcompartment identities (Supplemental Fig. S2E). Furthermore, we observed discrete *PAX6*^*+*^ and *EOMES*^*+*^ clusters (Supplemental Fig. S2F,G), including glut_progenitors and GN/UBC_progs, which were enriched for the PRC.RL^SVZ^ and UBC.RL^SVZ^ signatures, respectively (Fig. 2C). Interestingly, RNA velocity vector fields predict that glut_progenitors arose from the early_RL/NSC cluster (Supplemental Fig. S2H), which expresses the *PRTG*^+^, *SOX2*^+^, *MYC*^high^, and *NESTIN*^low^ signature also associated with G3 cells of origin (Fig. 2B).

We therefore grouped clusters into G3 and G4 trajectories and calculated differential gene expression over pseudotime (Fig. 2D), revealing sequential expression of neuronal genes associated with early lineage commitment and maturation (Supplemental Fig. S2I). We observed an earlier high expression of the G3-associated genes *PRTG*, *OTX2*, and *MYC* during sustained *SOX2* expression in the G3 trajectory, in line with G3 cells of origin harboring stem and early progenitor phenotypes (Fig. 2D). In contrast, in the G4 trajectory, MB-associated genes *EOMES* and *PAX6* exhibited high expression relatively later in pseudotime (after *SOX2* loss and before upregulation of the mature glutamatergic marker *SLC17A6*), highlighting a more committed progenitor cell population (Fig. 2D).

Finally, we used immunohistochemistry (IHC) to validate the presence of putative G3/4 MB cells of origin in CbOs and evaluated their spatial distribution. EOMES^+^Ki67^+^ cells reminiscent of the human-specific G3/4 MB-susceptible progenitors were located predominantly within the neural rosettes (Fig. 2E,F). In contrast, PRTG^High^;SOX2^+^;NESTIN^Low^ cells associated with G3 MB were found predominantly outside of neural rosettes (Fig. 2G,H), suggesting a spatial complexity to glutamatergic NSC/progenitor cells in CbOs with differing MB cell of origin signatures.

Taken together, our data provide the first evidence that CbOs harbor both G3 and G4 MB cells of origin. Furthermore, our findings may help to unify competing theories of G3 cells of origin by suggesting that they represent partially overlapping populations corresponding to subtly different stages along a shared G3 lineage of origin.

### Genetic engineering of MB cell of origin populations in CbO models neoplastic transformation

**Figure 3.**
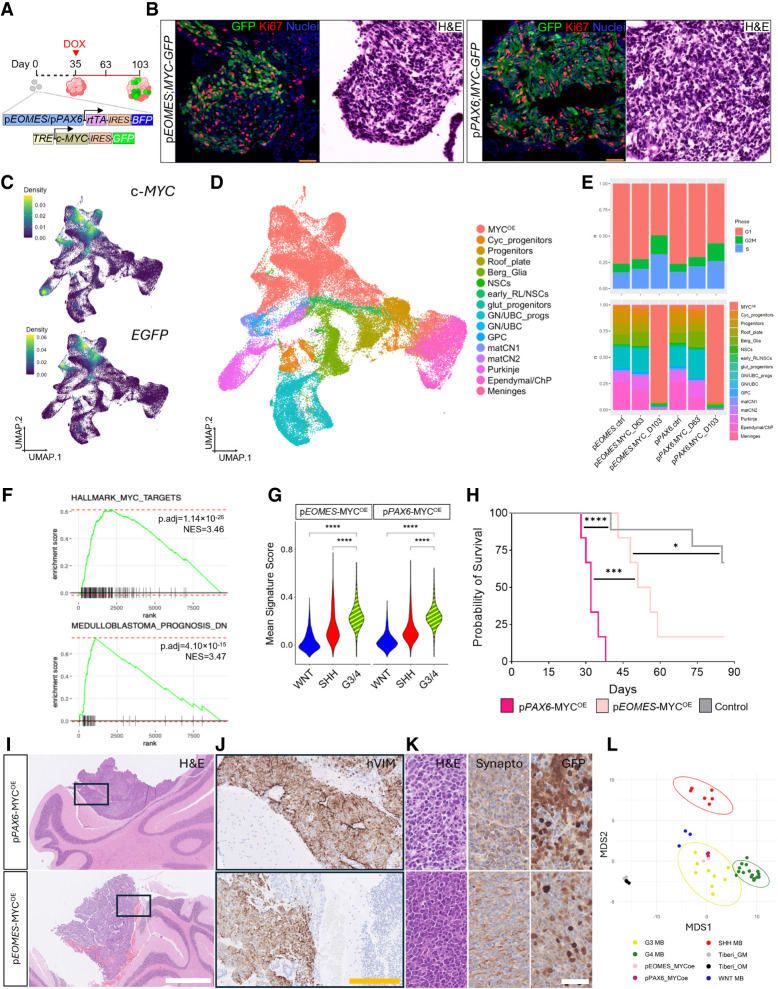
Engineered medulloblastoma lineages of origin display features of MB initiation upon c*-MYC* overexpression and form MB tumors in vivo. (*A*) Schematic of cerebellar organoid (CbO)-editing strategy (see the Materials and Methods). (*B*) Representative immunohistochemistry staining for GFP (green) and Ki67 (red) and H&E staining for CbO_19 collected at day 63 of differentiation for DOX-induced p*EOMES;MYC-GFP* and p*PAX6;MYC-GFP* organoids. Scale bar, 50 µm. (*C*) UMAP plot of scRNA-seq data from DOX-induced p*EOMES;MYC-GFP* and p*PAX6;MYC-GFP* at days 63 and 103 and control DOX-induced p*EOMES* and p*PAX6* CbOs at day 63 of differentiation, with c-*MYC* (*top*) and *EGFP* (*bottom*) nebulosa expression density plotted. Pooled samples of *n* = 3 CbOs per condition. (*D*) UMAP plot shown in *C*, with annotations transferred from reference D35/D49 CbOs and the *MYC*-overexpressing (MYC^OE^) cluster annotated. (*E*) Bar plot of individual samples shown in *D*, scored for cell cycle phase (*top*) and relative proportions of each annotated cluster per sample (*bottom*). (*F*) Gene set enrichment plot of the HALLMARK_MYC_TARGETS (*top*) and POMEROY_MEDULLOBLASTOMA _PROGNOSIS_DN (*bottom*) gene sets in the MYC^OE^ cells compared with D35/49 G3/4 lineages of origin (Fig. 2D). (*G*) Violin plots showing mean signature scores for WNT, SHH, or G3/4 MB subgroups (Hovestadt et al. 2019) in MYC^OE^ cells. Wilcoxon test: (****) *P* < 0.0001. (*H*) Kaplan–Meier survival of DOX-treated mice following orthotopic xenograft with p*PAX6*-MYC^OE^ (*n* = 6), p*EOMES*-MYC^OE^ (*n* = 6),^,^ or control (*n* = 9) D103 CbOs. (*) *P* < 0.05, (***) *P* < 0.001, (****) *P* < 0.0001. (*I*) Representative H&E staining of the cerebella of mice xenografted with MYC^OE^ CbOs. Scale bars, 1 mm. (*J*) Representative IHC staining showing human Vimentin (hVIM) expression in the cerebella of mice xenografted with MYC^OE^ CbOs. Scale bars, 200 µm. (*K*) Representative H&E (*left*), synaptophysin (*middle*), and GFP (*right*) staining of hVIM-positive tissue areas. Scale bar, 50 µm. (*L*) Multidimensional scaling (MDS) clustering of the top 1000 most variable features of DNA methylation profiles of MYC^OE^ tumors and previously published bulk-engineered organoids (GM and OM) and patient MB samples (Ballabio et al. 2020).

Putative G3/4 MB cells of origin were identified through bioinformatic comparative analysis of MB and developing cerebellar samples; however, previous modeling in mice or CbOs has not spatiotemporally restricted oncogene activation to these cells. To functionally validate the susceptibility of these putative lineages of origin to neoplastic transformation, we used a Tet-inducible system driven by the promoters of *PAX6* or *EOMES* to overexpress the c-*MYC* oncogene, a hallmark of aggressive G3 MB also detected in a subset of G4 MB (Northcott et al. 2012; Schwalbe et al. 2025); GFP expression driven by an *IRES* sequence was also included (Fig. 3A). Both transcription factors are known to have a role in MB (Jones et al. 2012; Zagozewski et al. 2020), with PAX6 expressed strongly in the G3 lineage of origin, while *EOMES* is expressed in both G3 and G4 lineage of origin progenitor clusters at D35 (Fig. 2B; Supplemental Fig. S2F,G). c-*MYC* overexpression was induced by doxycycline (DOX) at D35, and GFP was monitored over time in CbOs, followed by harvesting for histological and scRNA-seq analysis at D63 and D103 (Fig. 3A). Foci of c-MYC-GFP^+^ cells were observed specifically in DOX-induced *pEOMES;MYC-GFP* or *pPAX6;MYC-GFP* CbOs as early as 4 days after treatment, which expanded over time (Supplemental Fig. S3A). At D63, CbOs showed clusters of highly proliferative GFP^+^Ki67^+^ cells, which corresponded to areas of densely packed hyperchromatic and mitotically active cells reminiscent of MB cellular morphology (Fig. 3B).

ScRNA-seq data from engineered CbOs were annotated by transferring cell type labels from our D35/D49 data set (Fig. 2A) and queried for c-*MYC* and *GFP* expression (Fig. 3C). This identified seven c-*MYC-*overexpressing (MYC^OE^) clusters (1, 2, 4, 5, 10, 13, and 22) that expressed both c-*MYC* and *GFP* and were almost exclusively composed of cells from DOX-induced *pEOMES;MYC-GFP* or *pPAX6;MYC-GFP* CbOs (Fig. 3D,E; Supplemental Fig. S3B,C). MYC^OE^ clusters demonstrated a stem/progenitor-like state (Supplemental Fig. S3C,D), in keeping with the transformation of MB cells of origin being linked to their stalled differentiation and failed cell cycle exit (Hendrikse et al. 2022). Consistent with this finding, D103 samples contained more proliferating cells and had drastically expanded MYC^OE^ populations (Fig. 3E).

GSEA on MYC^OE^ clusters revealed the expected enrichment of MYC targets alongside a significant enrichment of a gene set corresponding to poor MB prognosis (Fig. 3F). Scoring cells for patient-derived MB gene signatures confirmed that MYC^OE^ clusters were more similar to G3/4 MB than WNT or SHH subgroups (Fig. 3G) and were strongly enriched for G3 MB gene expression (Supplemental Fig. S3E). Upon comparing the seven MYC^OE^ clusters, we observed differential enrichment for cycling-like, stem-like, and glutamatergic progenitor-like identities in both models, likely reflecting cells at differing stages of early tumor initiation and evolution (Supplemental Fig. S3F). Interestingly, inferred copy number analysis indicated that by D103, p*PAX6*-MYC^OE^ cells acquired amplification in chromosome 12 (Supplemental Fig. S4A), which has been shown to simultaneously arise upon c-*MYC* amplification in clonal modeling of G3 MB tumor onset (Okonechnikov et al. 2025), providing further evidence of early neoplastic transformation.

We further investigated the methylation profiles of sorted GFP^+^-MYC^OE^ cells as compared with BFP^+^ lineage of origin controls, where RT-qPCR confirmed the activity of each promoter-driven system and specific overexpression of c-*MYC* in GFP^+^ samples (Supplemental Fig. S4B). Chromosome 12 amplification was also confirmed in these p*PAX6-*MYC^OE^ D103 cells (Supplemental Fig. S4C; Capper et al. 2018). We observed broad methylation changes by D103 upon c-*MYC* overexpression (Supplemental Fig. S4D), in keeping with a high degree of epigenetic dysregulation observed in MB tumors (Badodi and Marino 2022). Genes with hypomethylated promoters in D103 MYC^OE^ samples included G3/4 MB markers *TRIM58*, *GABRB3*, and *ERBB2* as well as enhanced hypomethylation of the G3 MB drivers *OTX2* and *GFI1* in the p*PAX6-*driven system. Importantly, we observed progressive hypermethylation in the promoters of the *TP53* and *CDKN2A* tumor suppressor genes, highlighting a potential susceptibility of MYC^OE^ cells to tumor initiation. Gene sets showing a concordant differential regulation of DNA methylation and transcription were enriched for pathways involved in dysregulation of glutamatergic neuronal differentiation and apoptosis in MYC^OE^ cells, highlighting the stalled differentiation and aberrant development of these cells, as well as the SMAD and TGFβ signaling pathways implicated in G3 MB progression (Supplemental Fig. S4E; Henderson et al. 2017; Morabito et al. 2019).

We next set out to functionally validate the transformation of MYC^OE^ cells. We first established that FACS-sorted GFP^+^ D103 MYC^OE^ cells could be serially passaged as cocultures in naive CbOs (Supplemental Fig. S5A) to permit longer in vitro 3D growth and profiled them by scRNA-seq. Cocultured MYC^OE^ cells were distinguished from the naive CbO cells based on inferred copy number alterations and *GFP* expression and retained high G3 MB signature scores (Supplemental Fig. S5B,C). Interestingly, while we again detected chromosome 12 amplification in a subset of p*PAX6*-MYC^OE^ cells, we observed new chromosomal alterations in the p*EOMES*-MYC^OE^ cells, including chromosome 17 amplification, a hallmark of G3/4 MBs (Supplemental Fig. S5B,C; Sharma et al. 2019). As expected, MYC^OE^ cells expressed significantly higher c-*MYC* levels than naive CbO cells (Supplemental Fig. S5D), but notably, MYC^OE^ cells that had acquired chromosomal alterations had reduced c-*MYC* levels (Supplemental Fig. S5E). We therefore tested whether modeled MB cells had begun to lose their reliance on c-*MYC* by removing DOX and conducting extreme limiting dilution assays (ELDAs). MYC^OE^ cells demonstrated significantly greater sphere formation compared with cell of origin controls; however, no significant difference in sphere formation or expression of the proliferation marker *KI67* was observed upon DOX withdrawal (Supplemental Fig. S5F,G). Importantly, analysis of GFP intensity in MYC^OE^ cells confirmed the deactivation of the Tet-On system upon DOX withdrawal (Supplemental Fig. S5H). These data support our epigenomic and transcriptomic analysis, indicating that sustained induction of the engineering system drives global deregulation of MB cells of origin beyond c-*MYC* overexpression alone, including the acquisition of additional mutations and phenotypes consistent with MB.

Finally, D103 p*PAX6-*MYC^OE^ or p*EOMES-*MYC^OE^ CbOs were orthotopically xenografted into neonatal mouse cerebellum to assess their tumorigenicity and to compare 3D in vitro serial passage with in vivo expansion. Mice engrafted with both MYC^OE^ systems demonstrated significantly lower survival compared with controls, with p*PAX6-*MYC^OE^ mice showing the poorest overall survival of <40 days (Fig. 3H), similar to that of *MYC*-amplified G3 MB PDX models (Hermans and Hulleman 2020). Histological analysis of MYC^OE^ mouse cerebella demonstrated highly cellular, hyperchromatic, and proliferative embryonic tumors, in keeping with MBs, which were positive for human Vimentin (Fig. 3I,J), confirming their CbO origin, and for synaptophysin (Fig. 3K), as expected in MBs (Schwechheimer et al. 1987). MYC^OE^ tumors exhibited heterogeneous GFP positivity (Fig. 3K), in keeping with the loss of DOX dependency of these cells as they transform. No MB tumors were detected in the control mice. Interestingly, unlike their in vitro serially passaged counterparts, D103 MYC^OE^ cells acquired no additional CNV mutations upon xenograft (Supplemental Fig. S5I) despite being profiled at a similar time point (∼40–50 days to mouse collection and 57 days of serial passage to scRNA-seq submission). Importantly, analysis of DNA methylation profiles demonstrated closer clustering of MYC^OE^ tumors with G3 MB patient tumors compared with previously published bulk-engineered organoids (Ballabio et al. 2020), highlighting the importance of specifically targeting human-specific cells of origin for MB modeling (Fig. 3L).

Taken together, we show that CbOs are suitable to model neoplastic transformation of putative fetal G3/4 MB lineages of origin, leading to their stalled development and transcriptional changes consistent with epigenetic dysregulation. c-*MYC* overexpression in the p*PAX6-*targeted system, enriched in the G3 lineage of origin, gave rise to MB tumors with the poorest survival, in keeping with highly aggressive *MYC-*amplified G3 MB.

### CbOs sustain proliferation and invasion of cocultured medulloblastoma cells and recapitulate in vivo drug testing responses

**Figure 4.**
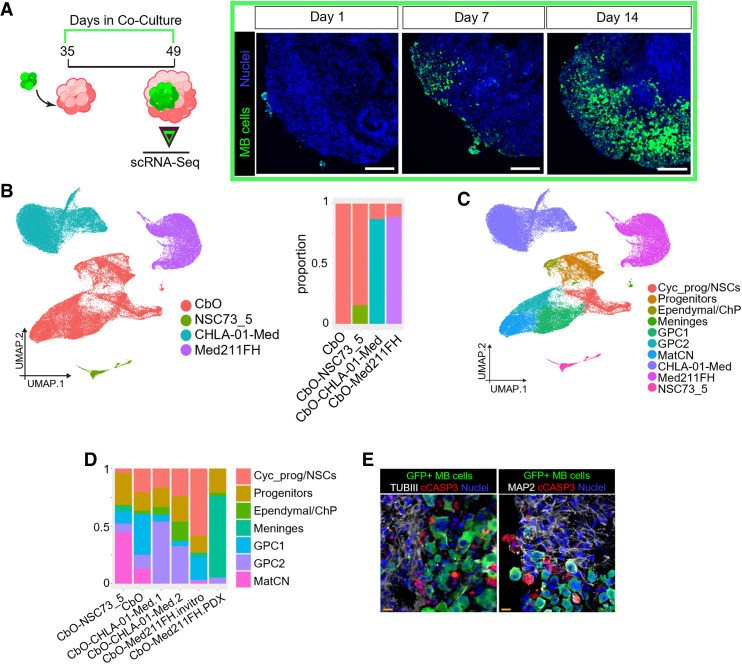
CbOs sustain cocultured medulloblastoma cell proliferation and invasion. (*A*, *left* panel) Schematic of the protocol used to coculture cerebellar organoids (CbOs) with medulloblastoma (MB), showing CbO-MB samples used for scRNA-seq. (*Right* panels) Representative immunofluorescence staining of GFP^+^ CHLA-01-Med MB cells upon 1, 7, and 14 days of coculture with CbO_61. Scale bars, 100 µm. (*B*, *left*) UMAP plot of scRNA-seq data from D49 control CbO_61, CbO-Med211-FH, CbO-CHLA-01-Med, and control CbO-NSC73_5 cocultures, colored by genotype. (*Right*) Bar plot showing the proportion of cells from each genotype in the different experimental models. Pooled samples of *n* = 3 CbOs/cocultures per condition. (*C*) UMAP plot of scRNA-seq data shown in *B*, colored by transferred cell type. (*D*) Bar plot showing the proportion of CbO cell types only in each sample submitted for scRNA-seq analysis. (*E*) Representative immunohistochemistry of CbO-Med211FH cocultures stained for GFP (green), cCASP3 (red), and TubIII (white; *left*) or MAP2 (white; *right*). Scale bars, 10 µm.

Next, we set out to assess whether CbOs could provide a physiologically relevant 3D environment for sustaining the growth of human MB cells (CbO-MBs). We cocultured GFP-tagged G3/4 MB cell lines (CHLA-01-Med, CHLA-01R-Med, and ICb1299) as well as G3 MB patient-derived primary cells (Med211-FH) maintained as PDXs or grown in vitro with D35 CbOs (Fig. 4A). All MB lines successfully attached and infiltrated the CbOs with considerable cell growth while maintaining an MB morphology as assessed at 14 days of coculture (Fig. 4A; Supplemental Fig. S6A–F). We observed positivity for the proliferation marker Ki67 in ∼30% of GFP^+^ MB cells (Supplemental Fig. S6G), thus confirming that CbOs can sustain MB cell proliferation and infiltration.

To explore the effect of MB cells on CbOs, we performed scRNA-seq on pooled CbO-MB samples (CHLA-01-Med or Med211-FH) after 14 days of coculture as well as on control CbOs cocultured with cerebellar NSCs (CbO-NSCs). Louvain clustering and inferred copy number analysis identified CHLA-01-Med and Med211-FH MB cells (Fig. 4B; Supplemental Fig. S6H) expressing neuronal-specific (*SIX3*/*APOE* and *NEUROD1*/*BCAT1*, respectively) and MB-specific (*HMGA1*, *MYC*, and *YBX3*) markers (Supplemental Fig. S6I). Cocultured NSCs could be distinguished by their separation in the UMAP and expression of stemness markers (*SOX2/9*) (Fig. 4B; Supplemental Fig. S6I). In keeping with the highly proliferative properties of tumor cells, MB cells represented >80% of the sequenced CbO-MB cells as compared with NSCs forming ∼20% of CbO-NSCs (Fig. 4B). We next identified CbO-specific cell types in CbO-MBs, annotating clusters based on label transfer, using our noncocultured organoid as reference (Fig. 4C; Supplemental Fig. S6J,K). Strikingly, a depletion of mature cerebellar neurons (MatCNs) was evident in all CbO-MB cocultures but absent in naive CbO or CbO-NSC controls (Fig. 4D). Selective negative impacts on neuronal populations have been shown in other brain tumor organoid cocultures (Fan et al. 2024; Prior et al. 2024); we therefore reasoned that MB coculture was likely causing neuronal death. Using IHC, we validated the presence of apoptotic neurons (cCASP3^+^ and MAP2^+^/TubIII^+^) proximal to GFP^+^ MB cells at the periphery of CbO-MBs, away from the apoptotic/necrotic CbO core, which were not present in CbO-NSC controls (Fig. 4E; Supplemental Fig. S6L).

**Figure 5.**
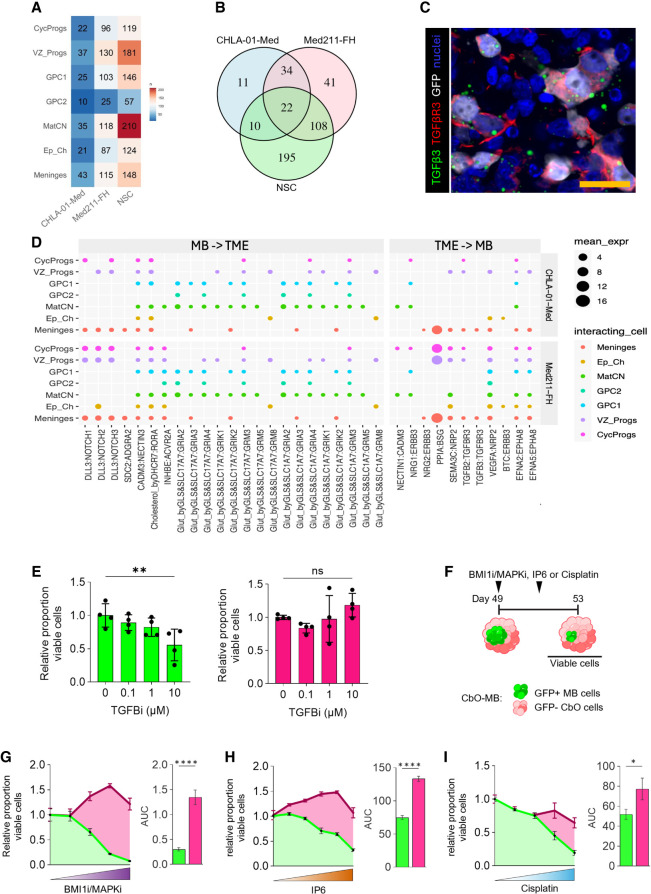
CbO-MB cultures are an effective tool for dissecting tumor–microenvironment interactions and for preclinical drug testing. (*A*) Heat map showing the total number of predicted significant receptor–ligand interactions between cocultured CbO-Med211-FH, CbO-CHLA-01-Med, and control CbO-NSC73.5 cells and CbO cell populations. (*B*) Venn diagram showing the shared significant receptor–ligand pairs modeled between cocultured CbO-Med211-FH, CbO-CHLA-01-Med, and CbO-NSC73_5 and CbO_61 cell types. (*C*) Immunohistochemistry of CbO-CHLA-01-Med cocultures stained for TGFβ3 (green), TGFβR3 (red), and GFP (gray). Scale bar, 20 µm. (*D*) Dot plot showing 34 shared MB-specific receptor–ligand pairs identified by CellPhoneDB analysis interaction (Efremova et al. 2020). Pairs in *D* are separated by cell line with CbO-CHLA-01-Med (*top*) and CbO-Med211-FH (*bottom*) and by direction with MB cells as a source (*left*) or as a target (*right*). (*E*) Viable populations of CHLA-01-Med (*left*) and CbO (*right*) populations following treatment with TGFβ inhibitor for 14 days of coculture, normalized to vehicle. One-way ANOVA: (**) *P* < 0.01; *n* = 4 cocultures per condition. (*F*) Schematic of CbO-CHLA-01-Med cocultures treated with combination BMI1/MAPK inhibitors, inositol hexakisphosphate (IP6), or cisplatin. (*G*–*I*) Viability assays of GFP^+^ MB cells (green) and GFP^−^ CbO cells (pink) upon treatment with increasing concentrations of BMI1 and MAPK inhibitors (*n* = 9; *G*), IP6 (*n* = 5; *H*), or cisplatin (*n* = 8; *I*). Measurement of area under curve (AUC) to compare overall treatment response (see also Supplemental Fig. S6M,N). Graphs report mean ± SEM, unpaired *t*-test: (****) *P* < 0.0001, (*) *P* < 0.05.

Little is known about the MB tumor microenvironment (TME), in terms of both the cross-talk between MB cells and nonneoplastic neuronal lineages and the molecular regulators of this interaction, or about the contribution of the MB–TME interplay to treatment response and recurrence in patients (van Bree and Wilhelm 2022). To characterize the molecular events mediating MB–TME cross-talk, we modeled receptor–ligand (R/L) interactions in CbO-MB cocultures using CellPhoneDB (Efremova et al. 2020). Overall, we found that both CHLA-01-Med and Med211-FH cells had fewer R/L interactions with CbO cells than NSC controls (Fig. 5A), in keeping with MB's limited TME reactivity compared with other brain tumors (van Bree and Wilhelm 2022). Among the 34 cancer-specific R/L interactions shared between MB cells but absent in NSCs (Fig. 5B), we observed interactions between multiple members of the TGFβ family as well as its downstream targets, including ERBB3, with the colocalization of TGFβ3/R3 on MB cells validated by immunofluorescence (Fig. 5C,D). TGFβ signaling has previously been implicated in G3 MB growth and invasion (Morabito et al. 2019); however, the role of the TME in its paracrine activation is poorly understood. Treatment of CbO-MB with a TGFβ inhibitor during coculture led to a significant decrease in MB cells (Fig. 5E), demonstrating an important role for TME signaling in MB progression, with potential implications for future MB treatment.

Tumor organoid coculture systems offer a promising approach for drug screening due to their human tumor microenvironment and relative affordability and scalability when compared with PDX models (Jin et al. 2018; Azzarelli 2020). We therefore sought to investigate whether CbO-MB could be suitable as a drug testing tool. We previously demonstrated that targeted combination therapies extend the survival of mice xenografted with MB cells, recapitulating a specific molecular signature found in a proportion of MB tumors, whereby the polycomb group gene BMI1 and chromatin remodeling factor CHD7 are overexpressed and downregulated, respectively (BMI1^High^;CHD7^Low^). Treatment with BMI1 (PTC-209) and MAPK (PD325901) inhibitors demonstrated a synergistic, cytotoxic effect on BMI1^High^;CHD7^Low^ MB cells, while the phospho-inositol metabolic modulator IP6 exerted cytostatic antitumor efficacy (Badodi et al. 2021, 2022). Here, we cocultured GFP-tagged BMI1^High^;CHD7^Low^ CHLA-01-Med cells with D35 CbOs and treated CbO-MB at 14 days, a time point when a large population of proliferative MB cells was established (Fig. 4A,B; Supplemental Fig. S6G), with BMI1/MAPK inhibitors or IP6 for 4 days (Fig. 5F). We observed a significant decrease in GFP^+^ BMI1^High^;CHD7^Low^ MB cells upon both treatments and a concomitant increase in cCASP3 or decrease in Ki67, respectively, with no significant impact on CbO cells (Fig. 5G,H; Supplemental Fig. S6M–P). To benchmark CbO-MB against the current patient standard of care, we also treated the cocultures with cisplatin and confirmed a significant decrease in viable MB cells compared with CbOs (Fig. 5I). However, a significant decrease in CbO viability was found at higher cisplatin concentrations (Supplemental Fig. S6M,N), in keeping with the long-term side effects and sequelae associated with current MB treatment (Fouladi et al. 2008; Vieira et al. 2014).

Taken together, these data show that CbO-MBs sustain the invasion and proliferation of MB cells and can be used to dissect tumor–microenvironment interactions. Moreover, MB cells grown in 3D models recapitulate the treatment response observed in a preclinical in vivo model as well as in patients and can be a suitable drug testing tool.

### Group 3 MB tumors harbor a subset of cells with myogenic differentiation, associated with poor prognosis

**Figure 6.**
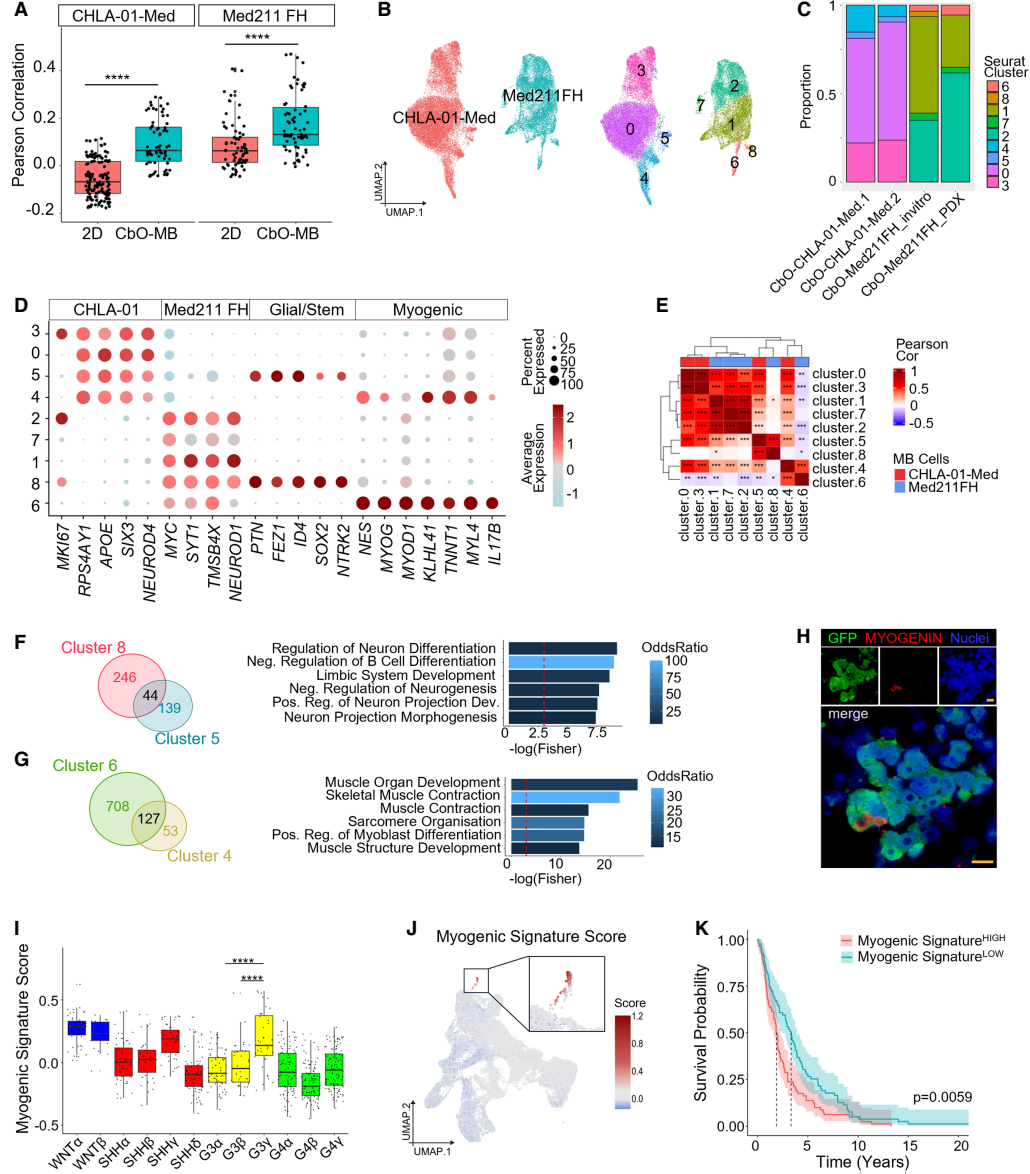
CbO-MB uncovers a subset of G3 MB cells with a myogenic transcriptional state. (*A*) Box plots showing Pearson correlations between MB cells grown in 2D (red) or 3D (blue) and published MB tumor samples (Hovestadt et al. 2019). One-way ANOVA: (****) *P* < 0.0001. (*B*) UMAP plot of scRNA-seq data from MB cells only from D49 CbO-Med211-FH and CbO-CHLA-01-Med cocultures (Fig. 4B), colored by genotype (*left*) and Louvain clusters (*right*). (*C*) Bar plot showing the proportion of MB cell clusters in each CbO-MB coculture. (*D*) Dot plot showing key marker genes used to identify MB cell cluster types in *B*. (*E*) Heat map showing Pearson correlation of MB cell clusters across the top 250 specific markers for each cluster. (*) *P* < 0.05, (**) *P* < 0.01, (***) *P* < 0.001. (*F*,*G*, *left*) Venn diagrams showing the intersection of CbO-Med211-FH (cluster 8 or 6) and CbO-CHLA-01-Med (cluster 5 or 4) cluster marker genes. (*Right*) Gene ontology enrichment bar plot of the 44 (*F*) or 127 (*G*) shared cluster marker genes. (*H*) Representative immunohistochemistry of CbO-Med211-FH cocultures stained for GFP (green) and MYOGENIN (red). Scale bar, 10 µm. (*I*) Box and whisker plot showing signature scores for the myogenic signature in different MB subtypes of the reference cohort of patient MB RNA expression data (Cavalli et al. 2017). Wilcoxon test: (****) *P* < 0.0001. (*J*) UMAP plot of CbO samples from c-*MYC* overexpression editing experiment (Fig. 3D; Supplemental Fig. S3B), scored for the myogenic signature. (*K*) Kaplan–Meier survival data of MB patients shown in *I*, split by myogenic high (>median; red) or myogenic low (<median; blue) signature score. Log-rank test: *P* = 0.0059.

To confirm that cocultured MB cells retained their subtype specificity, we scored computationally isolated MB cells for patient-derived MB gene signatures (Hovestadt et al. 2019), observing the expected enrichment in G3/4 metaprogram scores (Supplemental Fig. S7A). Similarly, when we correlated pseudobulk transcriptional profiles of a reference cohort of patient MB tumors (Hovestadt et al. 2019), CHLA-01-Med fell within an intermediate subcluster containing both G3 and G4 primary tumors, while Med211-FH clustered with G3 tumors only (Supplemental Fig. S7B). Computational label transfer of G3 and G4 tumor scRNA-seq profiles onto cocultured MB clusters confirmed strong G3 enrichment in Med211FH cells and mixed G3/4 enrichment in CHLA-01-Med cells (Supplemental Fig. S7C). Studies of glioblastoma have shown that tumor cells more closely recapitulate their parental tumors when cocultured in brain organoids as compared with 2D models (Pine et al. 2020). We therefore correlated expression profiles of each MB line cocultured with CbOs or in 2D with patient tumor samples (Hovestadt et al. 2019; Badodi et al. 2021). Importantly, we observed a significantly higher correlation in all cocultured MB cells compared with their 2D cultured counterparts (Fig. 6A), suggesting that CbO-MBs better recapitulate in vivo primary tumors.

To evaluate MB cell heterogeneity, we performed Louvain clustering and correlated cluster marker signatures to each other and to references of the developing cerebellum (Fig. 6B; Supplemental Fig. S7D). As expected, given the glutamatergic neuronal origins of G3/4 tumors, both cell lines were predominantly composed of clusters corresponding to GCP-like cells (clusters 1, 7, and 2 for Med211-FH and clusters 3 and 0 for CHLA-01-Med) and expressed genes, consistent with a neuronal phenotype (*SYT1*, *NEUROD1*, *SIX3*, and *NEUROD4*, respectively) (Fig. 6B–D; Supplemental Fig. S7D). We also identified two pairs of smaller cell states that were common to both MB lines: clusters 8 and 5 from Med211-FH and clusters 4 and 6 from CHLA-01-Med (Fig. 6C–E). Clusters 8 and 5 correlated with glial progenitors/NSCs developing cerebellar cell types (Supplemental Fig. S7D) and expressed stemness markers (*SOX2* and *NTRK2*) (Fig. 6D). GO enrichment of the 44 shared marker genes highlighted terms associated with neuronal development and radial glia differentiation (Fig. 6F). Clusters 6 and 4 showed a phenotype more consistent with immune reference cells and expressing immune-associated markers such as *IL17B* (Fig. 6D; Supplemental Fig. S7D). Further analysis of the 127 shared marker genes between clusters 6 and 4 revealed a strong enrichment of GO terms associated with myogenic development and differentiation (Fig. 6G). This included expression of key transcription factors governing myogenic differentiation (*MYOG*, *MYOD1*, and *HLHL41*) (Fig. 6D), with MYOGENIN^+^ MB cells confirmed by IHC in CbO-MB cocultures (Fig. 6H). RNA velocity vector fields suggest that myogenic cells represent a differentiation end point rather than a transitory transcriptional state (Supplemental Fig. S7E). Previous histological studies have reported aberrant myogenic differentiation of MB cells (Rattenberry et al. 2011; Crawford and Levy 2015), but the transcriptional state associated with such phenotypes has not been described previously. By scoring reference MB samples (Hovestadt et al. 2019) for our CbO-MB-derived myogenic state signature, we observed the highest mean score in the WNT subgroup, while G3 MB revealed a subset of cells scoring highest for our signature, suggesting heterogeneous expression (Supplemental Fig. S7F). Indeed, upon subsetting and reclustering G3 MB scRNA-seq samples, we observed two clusters, 4 and 6, that expressed this myogenic signature strongly with varying abundance across patient samples (Supplemental Fig. S7G,H). Scoring of the myogenic signature in a larger cohort of 763 MB samples (Cavalli et al. 2017) revealed a similar distribution across MB groups (Supplemental Fig. S7I). MB subtype analysis revealed high enrichment in WNT subtypes (both of which demonstrate high c-*MYC* expression due to WNT pathway activation) and the c-*MYC*-amplified group3_γ subtype (Fig. 6I). This led us to hypothesize that high c-*MYC* expression may drive this phenotype rather than CNVs or cells of origin, given the disparate myogenic enrichment across MB subgroups/subtypes. We therefore leveraged our MYC^OE^ CbO models, scored their scRNA-seq profiles for the myogenic signature, and found specific enrichment in MYC^OE^ cluster 22 (Fig. 6J) as well as enrichment for the muscle cell differentiation GO pathway (Supplemental Fig. S4E). Importantly, in reference data, we found a significant reduction of survival in samples scoring highly (greater than the median) for the myogenic signature (Fig. 6K) and an enrichment in recurrent samples (Supplemental Fig. S7J).

Overall, we confirmed that cocultured MB cells retain their subgroup specificity and identified a subset of MB cells exhibiting myogenic differentiation linked to c-*MYC* expression, associated with poorer prognosis in patients.

## Discussion

We have shown that CbOs faithfully recapitulate the developmental stages of the human cerebellum relevant for MB initiation and contain cell lineages enriched for gene signatures of G3 and G4 MB cells of origin. Genetic engineering of these cells induced histological and molecular features of MB onset and led to tumor formation in vivo. Moreover, coculture of patient-derived MB cells within the CbOs generates a 3D in vitro model that sustains MB cell proliferation and invasion, revealing TME mediators of MB progression and validating their utility as a drug testing tool.

Recent studies have shown the fundamental role of human-specific stem and progenitor cells in the development of G3 and G4 MB (Hendrikse et al. 2022; Smith et al. 2022; Visvanathan et al. 2024), and it remains unclear whether the spatial arrangement, timing, and differentiation of lineages pertinent to MB formation are faithfully conserved between humans and mice (Butts et al. 2024), highlighting the need for new human-specific models. Putative G3 and G4 MB progenitor cells of origin are generated in the RL^SVZ^ upon RL compartmentalization at 11 PCW and show a specific spatiotemporal pattern that peaks at ∼14 PCW. Comparative epigenetic analysis of our CbO model and human cerebellar tissue samples indicated a level of maturation coincident with this key developmental window. Previously published cerebellar organoid protocols observed a correlation between 2 month old organoid cell populations and RL cell clusters from human data sets (Chen et al. 2023) and a modest enrichment for the G4 cell of origin UBC.RL^SVZ^ signature in the GNP cluster of a 3 month old organoid (Smith et al. 2022). We also observed significant enrichment for RL^SVZ^ signatures in discrete cell clusters at D35–D49 of CbO maturation. Importantly, we identified specific cell clusters showing enrichment for UBC.RL^SVZ^ and PRC.RL^SVZ^ expression signatures; the latter was never observed before in organoid models. Recent work also showed that G3 MB originates from *PRTG*^+^*;SOX2*^+^*;MYC*^high^*;NESTIN*^*l*^^*ow*^ cells. We observed enrichment for these genes and the RL^VZ^ cerebellar compartment signature in the less differentiated NSC population in our CbOs, which immediately preceded the PRC.RL^SVZ^ -enriched cluster in pseudotime/RNA velocity trajectories. Thus, our data provide a potential interpretative frame to unify competing theories, suggesting that both putative G3 cells of origin are partially overlapping populations corresponding to subtly different stages along a shared G3 lineage axis. Our CbO model therefore provides an unparalleled opportunity for modeling the initiation of G3 and G4 MBs by enabling access to their putative human-specific cells of origin in a 3D experimental system.

c*-MYC* amplification has been robustly demonstrated as an oncogenic driver in G3 MB and has been reported in a small proportion of G4 MBs (Northcott et al. 2012, 2017; Schwalbe et al. 2025). Notably, oncogenic drivers of G4 MB are ill-defined or have been implicated in MB progression rather than in initiation (Okonechnikov et al. 2025). We therefore overexpressed c-*MYC* in G3 and G4 MB cells of origin at an MB-relevant developmental stage to functionally assess their susceptibility to neoplastic transformation. This generated clusters of highly proliferative cells overexpressing *c-MYC* that are stalled in their differentiation and demonstrate an increased correlation to G3/4 MB tumors with phenotypes consistent with transformation. Strikingly, p*PAX6-*driven *c-*MYC expression induced hypomethylation of G3 MB drivers *GFI1* and *OTX2* as well as chromosome 12 amplification by D103 in MYC^OE^ cells. The co-occurrence of c-*MYC* and chromosome 12 amplification has been inferred clonally in G3 MB tumors through single-cell multiomics profiling (Okonechnikov et al. 2025). We demonstrate here their role in the early stages of transformation of their cells of origin, in keeping with oncogene aberrations enabling the acquisition of tumor-defining CNV mutations. In agreement, both MYC^OE^ systems formed tumors upon orthotopic xenograft, with p*PAX6*-MYC^OE^ demonstrating the worst prognosis. Importantly, DNA methylation profiling showed that our MYC^OE^ xenografts cluster more closely with G3 MB tumors than previous models overexpressing c-*MYC* in combination with *OTX2* or *GFI1* in bulk cerebellar organoid xenografts (Ballabio et al. 2020), highlighting the importance of modeling MB tumors in a cell of origin-specific context.

Interestingly, a large subset of p*EOMES*-MYC^OE^ cells acquired chromosome 17 amplification upon extended in vitro coculture beyond D103, a feature typical of G3 and G4 MBs (Sharma et al. 2019). This alteration was not observed in the matched xenograft model profiled at a similar time point, most likely because the bulk methylation profiling of xenograft tumors does not allow the detection of clonally restricted CNVs. However, the possibility that additional microenvironmentally mediated human-specific mutational events contribute to early MB initiation and transformation should also be considered, and such events may require the human-specific developmental landscape provided by CbO models.

Recent bioinformatic analysis has demonstrated a dynamic transcription factor network in G3/4 MB, involving *PAX6*, *EOMES*, and *c-MYC*, with distinct regulatory mechanisms driving G3, G4, or intermediate tumor identities (Joshi et al. 2024). It is therefore possible that the differences that we observed between p*PAX6-* and p*EOMES-*driven MYC^OE^ models reflect distinct regulatory landscapes, resulting in increased susceptibility to G3 MB onset in p*PAX6* cells and enabling earlier acquisition of additional mutations and epigenetic features upon MYC^OE^, leading to poorer prognosis. In contrast, the chromosome 17 amplification acquired later in p*EOMES* cells transformation coincided with loss of dependency on induced c-*MYC* expression, raising the possibility that this system models distinct G3/4 MB subtypes.

There is an emerging consensus that while G3/4 MB subtypes are derived from distinct cells of origin, they exist along a transcriptional continuum (Williamson et al. 2022), likely reflecting closely related cells of origin and partially overlapping mutational landscapes. Our work provides a framework for future studies using CbOs to model drivers of other G3/4 subtypes to dissect the contributions of lineage, oncogene, and gene regulatory network to MB onset and the broad chromosomal aberrations characterizing these tumors. Indeed, most MB subtypes have different male:female incidence ratios (Sharma et al. 2019), which may be linked to their developmental origins and known differences in male and female fetal brain development (Spiers et al. 2015; Wheelock et al. 2019). Studies comparing MB initiation in CbOs derived from male and female EPSC lines may help uncover the mechanisms underlying these clinical observations. Similar opportunities for researchers to leverage CbOs extend to other cerebellar-derived tumors such as ependymomas (Taylor et al. 2006; Johnson et al. 2010). Overall, we show that CbOs represent a flexible experimental platform that enables the functional validation of comparative computational analysis of single-cell data sets from early fetal cerebellum with full-blown MB tumors. In particular, this tool enables the investigation of key events linking early tumor initiation to malignant progression.

In vitro growth of MB cells isolated from patients’ tissues has proven difficult for some MB subgroups, particularly G4; thus, the number of reliable MB cell lines for use in 2D in vitro cell culture or PDX assays is limited (Ivanov et al. 2016; Badodi et al. 2019). Moreover, in vitro expansion can lead to shifts in molecular subgrouping, an issue that is particularly relevant for G3/4 MB (Sharma et al. 2019; Williamson et al. 2022). We successfully performed CbO cocultures with established and patient-derived MB lines of G3/4 and G3 identities. We also show that MB cells maintained as PDXs can be grown as CbO cocultures, expanding the applicability of our model. This suggests that our 3D coculture model represents a valuable tool to grow and maintain MB cell lines by exposing tumor cells to cell-extrinsic signals from the adjacent CNS tissue. It remains important to test whether patient-derived MB cells that cannot currently be cultured in vitro can be supported in CbOs, particularly G4 MB cells for which in vitro models are lacking. Achieving this will require a dedicated pipeline enabling parallel xenografting and CbO coculture in a prospective clinical setting.

Importantly, we observed that MB cells in the 3D CbO-MB model retain an expression profile closer to that of patient tumors and demonstrated that MB growth is supported by TME interactions with CbOs, as has been demonstrated in coculture models for other brain tumors (Pine et al. 2023; Nicholson et al. 2024). Specifically, we demonstrated that the TGFβ pathway previously shown to be upregulated in MB is activated by TME signaling, providing an important insight given recent studies investigating TGFβ inhibition for MB treatment (Santhana Kumar et al. 2018; Morabito et al. 2019). Importantly, our CbO-MB model recapitulates the therapeutic response to both targeted treatment and clinically used chemotherapy agents, supporting its value as a platform for testing novel MB therapeutic approaches. Notably, compared with PDX models, CbO-MB models are faster, scalable, and cost-effective (Hermans and Hulleman 2020; Heinzelmann et al. 2024) while also enabling the evaluation of toxicity on human cerebellar cells, highlighting their potential use in precision medicine applications.

Analysis of G3/4 MB cells in 3D coculture identified a cluster displaying myogenic differentiation—a lineage previously observed histologically in patients’ samples (Rattenberry et al. 2011; Crawford and Levy 2015), particularly in a small proportion of tumors that do not classify within the four accepted WHO subgroups (Gupta et al. 2018). Scoring of patient MBs for our CbO-MB-derived myogenic signature revealed the highest enrichment in WNT and a subset of G3 patients, both characterized by high c-*MYC* expression. Consistent with these findings, our CbO MYC^OE^ models also contained a subpopulation of cells undergoing myogenic differentiation, indicating that this phenotype can arise early during MB onset. Moreover, myogenic differentiation was associated with significantly poorer prognosis and recurrence. Given the fidelity with which CbO-MB and CbO-derived MYC^OE^ cells recapitulate this myogenic subset, these models may provide a valuable tool to further investigate its clinical relevance in MB onset, progression, and treatment.

In conclusion, we have established a human cerebellar organoid model that enables access to human G3/4 MB cells of origin and functionally validates their susceptibility to neoplastic transformation. Moreover, CbOs sustain G3/4 MB cell growth in a coculture system, recapitulating drug responses, including those observed in patients, highlighting their potential as a scalable preclinical screening platform for precision medicine. Finally, CbO-MB and CbO-derived MYC^OE^ models enabled the identification of a myogenic signature linked to c-*MYC* expression and poor prognosis.

## Material and methods

### Cell culture

Full characterization of the two EPSC lines used in this study has been reported previously (Vinel et al. 2021). Briefly, EPSC19 and EPSC61 were derived from fibroblasts isolated from the healthy dura mater of two female patients harboring no somatic mutations and reprogrammed based on established protocols (Yang et al. 2017). EPSCs were cultured as described previously (Vinel et al. 2021) on plates coated with Geltrex (Life Sciences) in mTEsR plus basal media (Stem Cell Technologies). During routine passaging, cells were detached with 0.1 mM EDTA (Life Sciences) and replated with media supplemented with 10 µM Y-27632 dihydrochloride (PeproTech 1293823). CHLA-01-Med and CHLA-01R-Med MB cell lines purchased from ATCC were cultured in suspension in Gibco DMEM-F12 media (Life Technologies) supplemented with 20 ng/mL human recombinant FGF and 20 ng/mL human recombinant EGF (PeproTech), 2% B-27 supplement (Invitrogen), and 1% penicillin/streptomycin. ICb1299 MB cells obtained from Dr Xiao-Nan Li (Baylor College of Medicine, Texas Children's Cancer Centre) (Shu et al. 2008; Zhao et al. 2012) were cultured semi-adherently in Gibco DMEM (Life Technologies) supplemented with 10% fetal bovine serum (Gibco) and 1% penicillin/streptomycin (Life Sciences). Med211-FH cells tagged with GFP were cultured in Neurobasal Plus media (Life Sciences) supplemented with N2 (Bio-techne), B27 (Bio-techne), 75 µg/mL BSA, 2 µg/mL heparin (Stem Cell Technologies), 10 ng/mL human recombinant FGF, and 10 ng/mL human recombinant EGF (PeproTech). Human fetal cerebellar NSC73.5 lines were obtained from the Cancer Research UK-funded Glioma Cellular Genetics Resource (https://www.institute-genetics-cancer.ed.ac.uk/facilities/glioma-cellular-genetics-resource) and cultured as described previously (Conti et al. 2005).

All cell lines were confirmed mycoplasma-negative prior to experimental procedures and cultured in humidified incubators at 37°C with 5% CO_2_ until ∼80% confluent before passaging.

### Cerebellar organoid differentiation

Cerebellar organoids were cultured based on a previously published protocol (Muguruma et al. 2015; Silva et al. 2020), as shown in Supplemental Figure S1A. Full details are in the Supplemental Material.

### CbO engineering strategy

To induce c*-MYC* expression in putative MB lineages of origin within developing CbOs, three lentivirus constructs were designed utilizing the doxycycline-inducible Tet-On system (Gossen et al. 1995). To target specific CbO lineages, the *rtTA* sequence element and *BFP* tag were placed under the control of the *EOMES* promoter (p*EOMES*) or a *PAX6* minipromoter (Ple260) (Hickmott et al. 2016) designed for CNS expression (p*PAX6*). In addition, each construct contained the puromycin resistance gene under the control of a constitutively expressed promoter (*mPGK*), giving *pPAX6-rtTA-BFP_mPGK-PuroR* or *pEOMES-rtTA-BFP_mPGK-PuroR*. The third construct contained the *c-MYC* sequence and *GFP* tag under the control of the tetracycline-responsive element (*TRE*) as well as the blasticidin resistance gene under the control of *mPGK*, giving *TRE-c-MYC-GFP_mPGK-BlastR*. Lentiviral constructs were produced as described previously (Badodi et al. 2017) and titrated using Lenti-X GoStix Plus as per the manufacturer's instructions (Takara Bio). EPSC19 cells were infected with *pPAX6-rtTA-BFP_mPGK-PuroR* or *pEOMES-rtTA-BFP_mPGK-PuroR* (MOI = 1) and selected for 24 h with 1.25 µg/mL puromycin and then coinfected with *TRE-c-MYC-GFP_mPGK-BlastR* construct (MOI = 1) and selected for 48 h with 5 µg/mL blasticidin. Cerebellar organoid protocol was then begun as detailed above. From day 35, 2 µg/mL doxycycline was added to culture media, and CbOs were monitored for GFP expression. Two sets of controls were included: noninduced CbOs harboring the full engineering system (no doxycycline control) and CbOs derived from EPSCs infected with the *pPAX6-rtTA-BFP_mPGK-PuroR* or *pEOMES-rtTA-BFP_mPGK-PuroR* constructs only and treated with doxycycline from day 35 onward (doxycycline + control). On days 63 and 103, engineered CbOs were taken for scRNA-seq analysis or IHC (both detailed below) or dissociated and then FAC-sorted using a BD FACSAria III cell sorter, and DNA was extracted and submitted for EPICv2 array (outlined below). GFP-positive day 103 engineered cells were further maintained in vitro beyond this time point as cocultures, which were serially passaged for 57 days before submission for scRNA-seq (160 days total).

### CbO-MB coculture

Prior to coculture, G3/4 MB cell lines were infected with shCHD7 lentivirus containing GFP as described previously (Badodi et al. 2021). On day 35 of CbO culture, organoids were placed in 96 well ultralow-attachment U-bottomed plates (Corning) in a 1:1 mix of complete MB cell media and complete neurobasal media with 100,000 MB cells. For Med-211-FH cells previously cultured as PDXs only, cells were thawed, underwent mouse cell depletion using the Miltenyi mouse cell depletion kit (130-104-694) according to the manufacturer's instructions, and were seeded onto CbOs as described above. The following day, half of the media from each well was replaced with complete neurobasal media. On day 37, the entire contents of each well were transferred to a 24 well ultralow-attachment plate (Corning) with 1 mL of complete neurobasal media. On day 4, total media was replaced with complete neurobasal media to remove noninfiltrating cells, and organoids were placed back on the orbital shaker at 90 rpm in a humidified incubator at 37°C with 5% CO_2_. Cocultures were maintained for a total of 14 days from MB cell seeding before being taken for further analysis. For TME validation assays, the TGFβ pathway inhibitor SB431542 or DMSO vehicle was added to the media throughout 14 days of coculture, and then CbO-MB cells were dissociated and analyzed with FACS as described below.

### DNA/RNA extraction

For cell culture samples (CbOs, EPSCs, and Med211-FH cells), DNA and RNA were extracted using the RNA/DNA/Protein Purification Plus kit (Norgen 47700) following the manufacturer's protocol. For FFPE human cerebellar samples, initial deparaffinization and DNA extraction were performed using the FFPE RNA/DNA Purification Plus kit (Norgen 54300). Samples were incubated with proteinase K overnight at 37°C to increase yield, after which DNA extraction was performed as per the manufacturer's instructions. For fresh-frozen human cerebellar samples, extraction was performed using the RNA/DNA Purification Plus kit (Norgen 47700) as per the manufacturer's instructions.

### RT-qPCR

All cDNA synthesis was performed using the SuperScript III reverse transcriptase kit (Invitrogen) according to the manufacturer's protocol. Analysis of gene expression was carried out using the Applied Biosystems 7500 real-time PCR with SYBR Green PCR MasterMix (Applied Biosystems) according to standard protocols. All qPCRs were performed in triplicate with 10 ng of cDNA. Average *C*_*t*_ values for target genes were normalized to *ACTIN B* and *GAPDH* housekeeping genes before calculating fold change. The primers used are listed in Supplemental Table S1.

### Immunohistochemistry (IHC)

Organoids were collected and fixed in 4% PFA in PBS, dehydrated in 30% sucrose in PBS, and embedded in 7.5% gelatin and 10% sucrose in PBS. Blocks were then snap-frozen in isopentane at −55°C before cryosectioning at 15 µm. Prior to staining, sections were degelatinized in warm water before permeabilization in 4% normal donkey serum with 0.25% Triton-X in PBS for 2 h. Sections were then washed in PBS before overnight incubation at 4°C with primary antibodies diluted in 4% normal donkey serum in PBS. A list of primary antibodies and dilutions is in Supplemental Table S2. Sections were then washed three times in PBS, followed by incubation for 3 h at room temperature with corresponding Alexa fluor secondary antibodies at a 1:500 dilution in PBS. After a final three PBS washes, sections were mounted with coverslips using ProLong Gold antifade reagent with DAPI (Thermo Fisher Scientific P36931). Images were taken using a Zeiss 880 laser scanning confocal microscope with Zeiss Zen software v2.3.

### CbO-MB drug assays

On day 0 of drug assays, CbO-MB cultures were transferred to new 24 well ultralow-attachment plates (Corning) containing 500 µL of complete neurobasal media plus a drug (PTC-209/PD325901 inhibitors, cisplatin, or IP6) or vehicle (DMSO, DMSO, or PBS, respectively) and then placed back on an orbital shaker where they remained for the duration of the assay. A drug and media top-up was performed on day 2. On day 4, the entire content of each well was collected, dissociated into a single-cell suspension using the neural dissociation kit (MACS Miltenyi Biotec), stained with Zombie NIR fixable viability kit (1:300 dilution; BioLegend), and analyzed using a BD LSRII flow cytometer. GFP-expressing MB cells were detected with the blue excitation laser at 488 nm, while the Zombie NIR-positive dead cells were detected with the red excitation laser at 633 nm. All subsequent single-cell gating and analyses were performed in FlowJo v10.6.2.

### Dissociation of CbOs and CbO-MBs for scRNA-seq

Pooled samples of three independent CbOs were taken at days 35 and 49, CbO-MB samples were taken at day 49, and samples of engineered CbOs were taken at days 63 and 103. Organoids were dissociated into single-cell suspension using the neural dissociation kit (MACS Miltenyi Biotec). Debris removal was then performed according to the manufacturer's protocol (Miltenyi Biotec 130-109-398). Cells were resuspended in loading buffer (0.04% BSA in PBS), strained through a 70 µm cell strainer, and counted using a hemacytometer with trypan blue staining. At least 10,000 cells per sample were taken for processing using 10x Genomics kits (detailed below).

### RNA sequencing

Med211-FH RNA from cells cultured in vitro was sequenced at Novogene with paired-end, 150 bp sequence reads. Library preparation [mRNA and poly(A) enrichment] and sequencing were performed on the NovaSeq 6000 platform. Reads were quality-trimmed using Trim Galore! v0.6.5 and aligned to the reference genome GRCh38.release109 using STAR v2.7.9a (Dobin and Gingeras 2015). Reads were counted using the featureCounts function and annotated with ENSEMBL Homo_sapiens.GRCh38.109.gtf.

### DNA methylation analysis

All CbO and human cerebellar DNA samples underwent standard BSC conversion at the University College London Genomics Facility (University College London, Great Ormond Street Institute of Child Health) and were submitted for Illumina Methylation EPIC v2 array following standard protocols. Quality control was performed, and probe β-values were extracted from raw .idat files using the wateRmelon R package (Pidsley et al. 2013) under the reference genome hg38. Probes with detection *P*-values >0.05 were removed using pfilter(), as well as probes associated with sex chromosomes, mitochondrial chromosomes, and single-nucleotide polymorphisms. Adjusted Dasen normalization was then performed. Methodological details for downstream analyses, including (1) cerebellar organoid developmental staging, (2) differentially and variably methylated probe and region analysis, (3) comparison with reference data sets, (4) pathway analysis, and (5) scRNA-seq/DNA methylation integration, are included in the Supplemental Material.

### scRNA-seq analysis

Library preparation was performed at the Single-Cell Genomics Facility (University College London, Cancer Institute) using the Chromium Next GEM single-cell 3′ kit v3.1 (10x Genomics 1000268) along with the Chromium Next GEM Chip G single-cell kit (10x Genomics 1000120) according to the manufacturer's instructions. Pooled-sample libraries were sequenced with lane sequencing on a NovaSeq 6000 S4 (Novogene). The Cell Ranger 2.0.1 pipeline was used to align reads to the GRCh38 human reference genome and to produce count matrices for downstream preprocessing and analysis using the Seurat v5.0.1 R package (Hao et al. 2021). Methodological details for downstream analyses, including (1) UMAP calculation, (2) cell type annotation, (3) comparison with reference data sets, (4) gene signature scoring, and (5) cell lineage trajectory analysis, are included in the Supplemental Material. For receptor–ligand analysis, cell type metadata and count data for the top 15,000 most highly variable genes in the CbO-MB data set were input into CellPhoneDB v5(Efremova et al. 2020) and run using the statistical_analysis mode.

### Orthotopic xenograft

D103 p*PAX6*-MYC^OE^ (*n* = 6) or p*EOMES*-MYC^OE^ (*n* = 6) CbOs were dissociated as described for scRNA-seq sample submission and injected into NOD-SCID CB17-Prkdcscid/J mouse neonates’ right cerebellar hemispheres (2 mm lateral and 2 mm posterior to λ, 2 mm deep) with a microfine insulin syringe and needle at postnatal day 5–6. One-hundred-thousand cells were injected per animal in 2 µL of sterile PBS. Time point-matched control CbOs harboring p*EOMES* or p*PAX6* constructs only were also injected (*n* = 9), and doxycycline (0.2% in 1% sucrose in H_2_O) was administered to all mice via drinking water throughout the experiment. Mouse weight was monitored throughout, animals were sacrificed by neck dislocation upon signs of CNS pathology symptoms, and brains were harvested. Brains were fixed in 4% PFA in PBS for 24 h and then subsequently processed for histology and immunohistochemistry at the University College London IQPath Facility. Six out of six p*PAX6*-MYC^OE^ and five out of six p*EOMES*-MYC^OE^ mice developed MB tumors. Two out of nine control mice developed teratocarcinomas but no MB.

### Ethics

Ethical approval for the work on human cerebellar samples was obtained from BRAIN UK (reference no. 22/010). All animal procedures were performed in accordance with licenses held under the Home Office Guidelines Scientific Procedures Act 1986 (PP3112577).

### Data availability

The authors declare that all the data supporting the findings of this study are available here and in the Supplemental Material. The scRNA-seq and DNA methylation data sets generated in this study and processed data are available in the NCBI Gene Expression Omnibus database (GSE263652 and GSE263653, respectively) or from the corresponding authors upon reasonable request. Publicly available data sets used in the study were retrieved from relevant databases as outlined in the Materials and Methods.

### Code availability

The authors declare that no custom code was used in this study. A full description of analyses, including functions and packages used, is detailed in Materials and Methods. Specific code will be made available from the corresponding authors upon reasonable request.

## Supplemental Material

Supplement 1
