# Supplementary material for: Modeling medulloblastoma pathogenesis and treatment in human cerebellar organoids
Source: Genes Dev. 2026 Jun 1;40(11-12):936–55. doi: 10.1101/gad.353292.125 (PMC13224868; doi:10.1101/gad.353292.125)
Supplement: Supplement 1 [file Supplemental_Figures_Legends.pdf]

Supplementary Figures and Legends

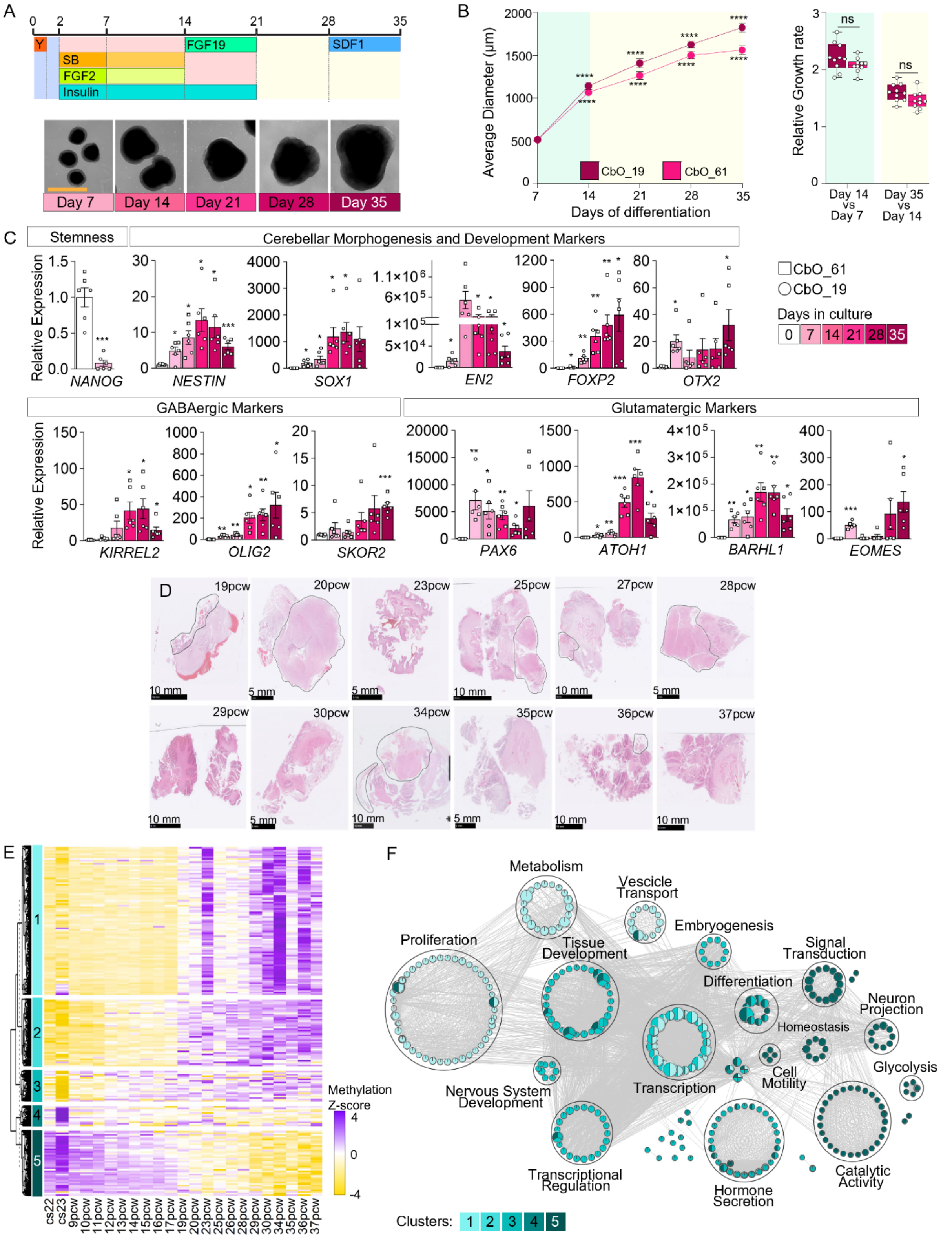

Supplementary Figure 1. CbO displays sustained growth and maturation. **(A)** Schematic of protocol used to generate and differentiate CbO, (see Methods, Y: Y-27632 Dihydrochloride and SB: SB431542) and representative brightfield pictures of CbO_61 upon 7, 14, 21, 28 and 35 days of differentiation. Scale bar = 1000 µm. **(B)** Diameter analysis of CbO obtained from two independent EPSC lines (CbO_19 and CbO_61) across 35 days of maturation (left) and analysis of relative growth comparing the diameter fold change of Day 14 to Day 7 or Day 35. Asterisks indicate p-values between given time point and Day 7, two-way ANOVA, ****$p<0.0001$, ns: not significant. n = 10 independent CbO average diameters from 3 batches per timepoint. **(C)** qPCR analysis showing quantification of cerebellar marker expression in CbO_19 and CbO_61 across 35 days of maturation. The relative RNA level was calculated as 2^(-dCT) values normalised to the EPSC baseline (Day 0). All graphs report mean ± SEM, Welch's unpaired t-test, *$p<0.05$, **$p<0.01$, ***$p<0.001$. Non-significant comparisons are not shown. Data points represent the average values from three CbO measurements taken from n = 3 independent batches of CbO at each time point. **(D)** H&E staining of FFPE samples from human foetal cerebellum. Non-cerebellar tissue excluded from the microdissection is circled. Developmental stage in post-conception weeks (pcw) and scale bars are shown. **(E)** Heatmap showing k-means clustering (k=5) of significant variable methylated genes across human foetal cerebellar development from Carnegie stage (cs) 22 to 37 post-conception weeks (pcw). Z-scores for average beta values of probes contained within each gene are plotted. **(F)** Bubble plot showing the top 100 ranked significant (adjusted p-value < 0.05) GO Biological Processes and Molecular Functions of genes from 5 clusters shown in B taken for pathway analysis in g:Profiler. Bubbles are coloured based on each cluster, and size is proportional to the number of genes in a specific GO term.

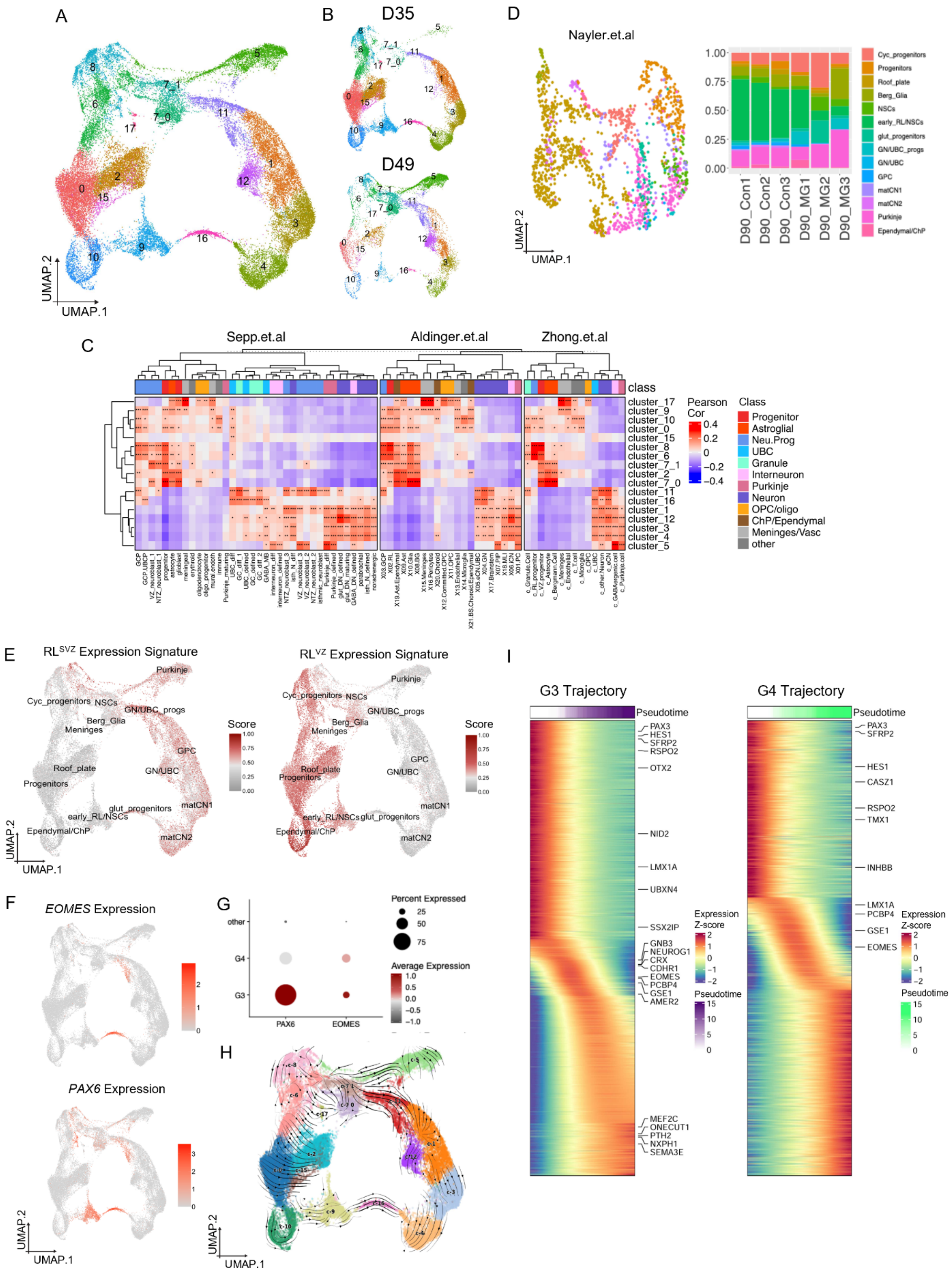
A
UMAP.2
UMAP.1
B
D35
D49
D
Nayler.et.al
1.00
0.75
0.50
0.25
0.00
D90_Con1
D90_Con2
D90_Con3
D90_MG1
D90_MG2
D90_MG3
Cyc_progenitors
Progenitors
Roof_plate
Berg_Glia
NSCs
early_RL/NSCs
glut_progenitors
GN/UBC_progs
GN/UBC
GPC
matCN1
matCN2
Purkinje
Ependymal/ChP
C
Sepp.et.al
Aldinger.et.al
Zhong.et.al
class
Pearson Cor
Class
Progenitor
Astroglial
Neu.Prog
UBC
Granule
Interneuron
Purkinje
Neuron
OPC/oligo
ChP/Ependymal
Meninges/Vasc
other
E
RLSVZ Expression Signature
RLVZ Expression Signature
Score
F
EOMES Expression
PAX6 Expression
G
Percent Expressed
Average Expression
other
G4
G3
PAX6
EOMES
H
I
G3 Trajectory
G4 Trajectory
Pseudotime
Expression Z-score
PAX3
HES1
SFRP2
RSPO2
OTX2
NID2
LMX1A
UBXN4
SSX2IP
GNB3
NEUROG1
CRX
CDHR1
EOMES
PCBP4
GSE1
AMER2
MEF2C
ONECUT1
PTH2
NXPH1
SEMA3E
CASZ1
TMX1
INHBB

Supplementary Figure 2. CbO scRNA-Seq profiles demonstrate similar cell types and lineages to the developing human foetal cerebellum.
(A) UMAP plot of scRNA-Seq data from CbO samples at Day 35/49 of differentiation, identifying 17 clusters and sub clustering of cluster 7 into 7_0 and 7_1. Low quality hypoxic clusters 13 and 14 not shown. **(B)** UMAP plot of scRNA-Seq data shown in A, split by Day 35 (left) and Day 49 (right) timepoints. **(C)** Heatmap showing Pearson correlation of CbO D35/49 clusters identified in K to clusters annotated by cell type of three independent scRNA-seq datasets of the human developing foetal cerebellum (Aldinger et al. 2021; Zhong et al. 2023; Sepp et al. 2024). Pearson correlation *p<0.05, **p<0.01, ***p<0.001. **(D)** UMAP showing clustering of Nayler et al cerebellar organoid scRNA-seq data (left) with CbO cluster annotation labels transferred to each sample in their dataset (right) (Nayler et al. 2021). **(E)** UMAP of Day 35/49 CbO showing expression score for the RL^{SVZ} (left) and RL^{VZ} (right) gene signature (Smith et al. 2022). **(F)** UMAP of Day 35/49 CbO showing ALRA-imputed expression score for *EOMES* (upper) and *PAX6* (lower). **(G)** Dot plot showing *EOMES* and *PAX6* expression in defined G3 (early_RL/NSCs & glut_progenitors) and G4 (GN/UBC_progs)cells-of-origin (Figure 2D) and in other remaining CbO D35/49 clusters. **(H)** Day 35/49 CbO UMAP with RNA velocity streams shown, calculated in velocyto and scVelo (La Manno et al. 2018; Bergen et al. 2020). **(I)** Plots of z-scored gene expression through pseudotime within G3 and G4 trajectories defined in Figure 2D.

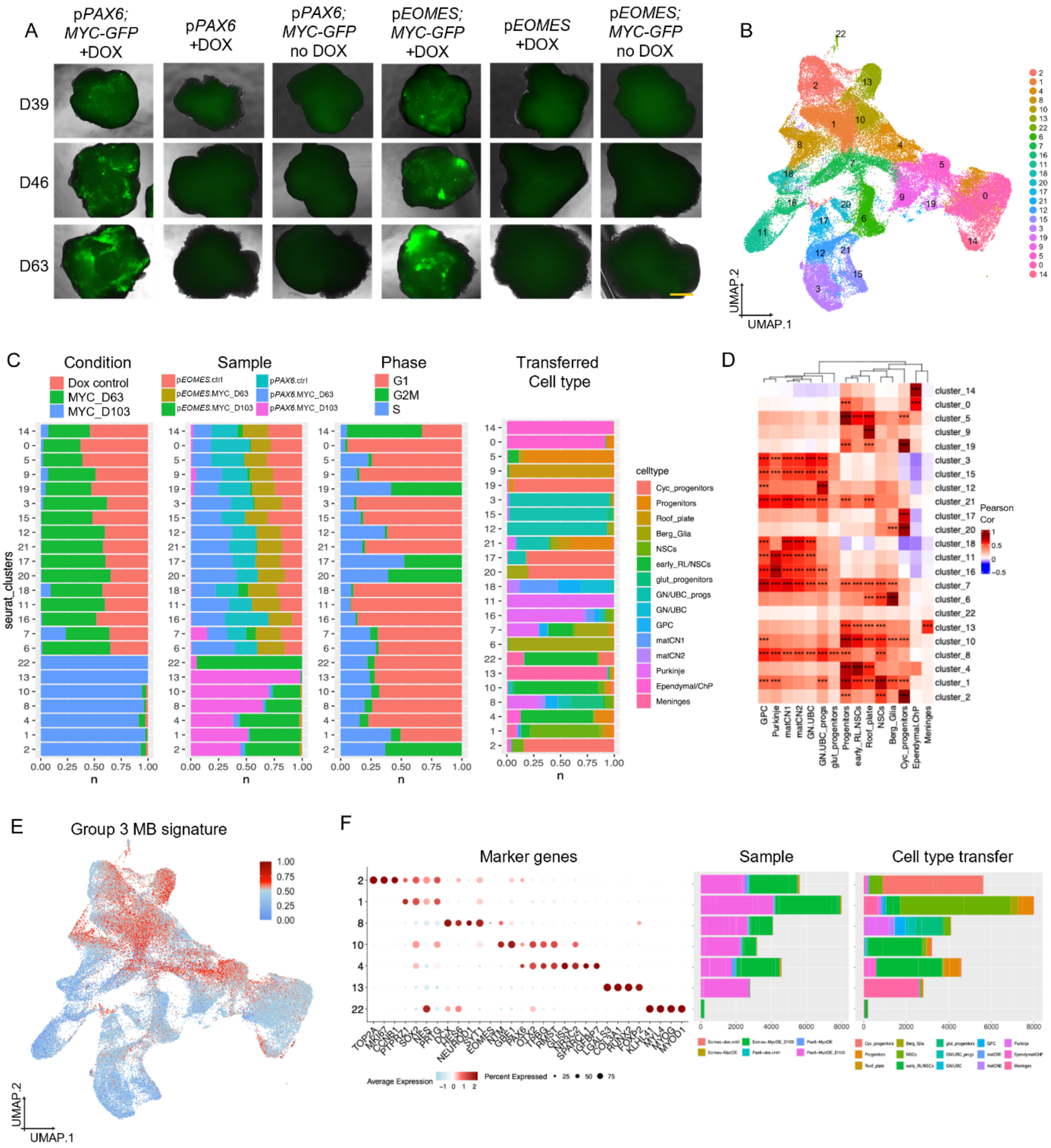
A
pPAX6; MYC-GFP +DOX
pPAX6 +DOX
pPAX6; MYC-GFP no DOX
pEOMES; MYC-GFP +DOX
pEOMES +DOX
pEOMES; MYC-GFP no DOX
D39
D46
D63
B
UMAP.2
UMAP.1
C
Condition
Dox control
MYC_D63
MYC_D103
Sample
pEOMES.ctrl
pEOMES.MYC_D63
pEOMES.MYC_D103
pPAX6.ctrl
pPAX6.MYC_D63
pPAX6.MYC_D103
Phase
G1
G2M
S
Transferred Cell type
celltype
Cyc_progenitors
Progenitors
Roof_plate
Berg_Glia
NSCs
early_RL/NSCs
glut_progenitors
GN/UBC_progs
GN/UBC
GPC
matCN1
matCN2
Purkinje
Ependymal/ChP
Meninges
seurat_clusters
n
D
Pearson Cor
E
Group 3 MB signature
F
Marker genes
Sample
Cell type transfer
Average Expression
Percent Expressed

Supplementary Figure 3. Cerebellar organoid editing strategy displays specificity to medulloblastoma lineages-of-origin.

(A) Representative images of CbO19 at Day 36, 46 and 63 of culture infected with different combinations of p*PAX6*, p*EOMES* or *MYC-GFP* editing constructs (see Figure 3A), with or without the addition of doxycycline (DOX) to organoid media from Day 35 of culture. Scale bar = 750 µm. **(B)** UMAP plot of scRNA-Seq data from DOX-induced p*EOMES;MYC-GFP* and p*PAX6;MYC-GFP* at Day 63 and 103 and control DOX-induced p*EOMES* and p*PAX6* CbOs at Day 63 of differentiation coloured by Louvain clusters. Pooled samples of n = 3 independent CbOs per condition. **(C)** Bar plots of samples described in B showing the relative proportion of Louvain clusters in each sample split by (left to right): condition, sample, cell-cycle phase and with cell types from reference D35/49 CbO annotated clusters transferred. **(D)** Heatmap showing Pearson correlation of Louvain clusters shown in B with reference annotated D35/49 CbO cell types. *** $p<0.01$. **(E)** UMAP plot shown in B, scored for a Group 3 MB gene signature (Vladoiu et al. 2019). **(F)** Analysis of the seven MYC^{OE} clusters showing marker gene expression (left), abundance in each sample (middle) and with cluster annotation labels transferred from D35/49 CbO (right).

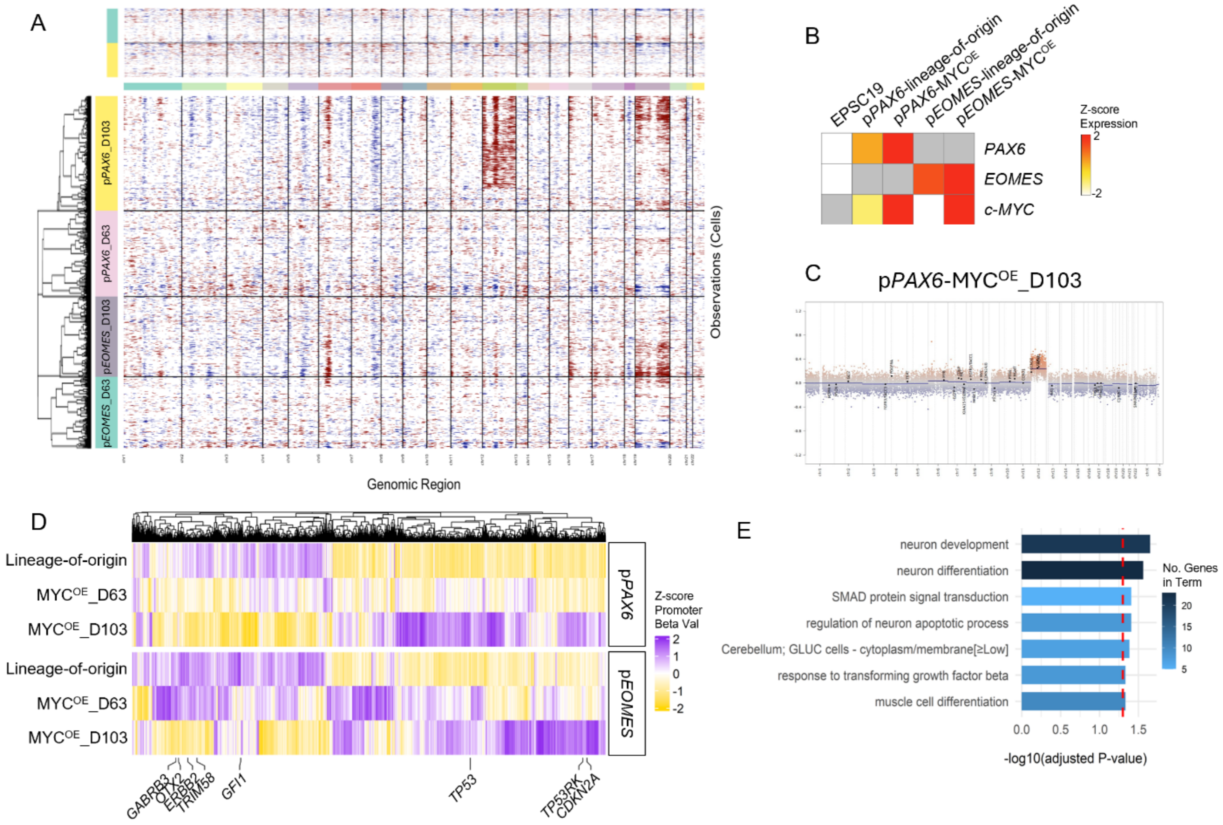
A
pPAX6_D103
pPAX6_D63
pEOMES_D103
pEOMES_D63
Observations (Cells)
Genomic Region
B
EPSC19
pPAX6-lineage-of-origin
pPAX6-MYCOE
pEOMES-lineage-of-origin
pEOMES-MYCOE
PAX6
EOMES
c-MYC
Z-score Expression
2
-2
C
pPAX6-MYCOE_D103
D
Lineage-of-origin
MYCOE_D63
MYCOE_D103
Lineage-of-origin
MYCOE_D63
MYCOE_D103
pPAX6
pEOMES
Z-score Promoter Beta Val
2
1
0
-1
-2
GABRB3
OTX2
ERBB2
TRIM58
GFI1
TP53
TP53RK
CDKN2A
E
neuron development
neuron differentiation
SMAD protein signal transduction
regulation of neuron apoptotic process
Cerebellum; GLUC cells - cytoplasm/membrane[≥Low]
response to transforming growth factor beta
muscle cell differentiation
No. Genes in Term
20
15
10
5
0.0
0.5
1.0
1.5
-log10(adjusted P-value)

Supplementary Figure 4. Day 103 MYC^{OE} cells display molecular features consistent with MB.

(A) Hierarchical clustering of inferred copy number profiles for scRNA-Seq samples of DOX-induced p*EOMES;MYC-GFP* and *pPAX6;MYC-GFP* at Day 63 and 103 with reference control CbO profile above. **(B)** Heatmap showing z-scored RT-qPCR expression of *PAX6*, *EOMES* and c-*MYC* for GFP-sorted MYC^{OE} cells (p*EOMES;MYC-GFP*, p*PAX6;MYC-GFP*) and BFP-sorted control lineage-of-origin samples. Z-scored -dCT values are plotted. **(C)** Genomic CNV profile for pPAX6-MYC^{OE}_D103 DNA sample (Capper et al. 2018). **(D)** Heatmap showing z-scored average beta values of probes contained within differentially methylated regions mapping to gene promoters between GFP-sorted MYC^{OE} cells (p*EOMES;MYC-GFP*, p*PAX6;MYC-GFP*) at Day 63 and 103 and BFP-sorted control lineage-of-origin samples. Key genes are annotated. **(E)** Bar plot of dysregulated GO biological process and human protein atlas pathways upon MYC^{OE} for genes with concordant methylation status and expression levels. Significant concordant genes were defined as hypomethylated/upregulated or hypermethylated/downregulated with an absolute beta difference > 0.1, absolute log2FC < 0.25 and p-adj < 0.05 between D103 MYC^{OE} cells and lineage-of-origin controls.

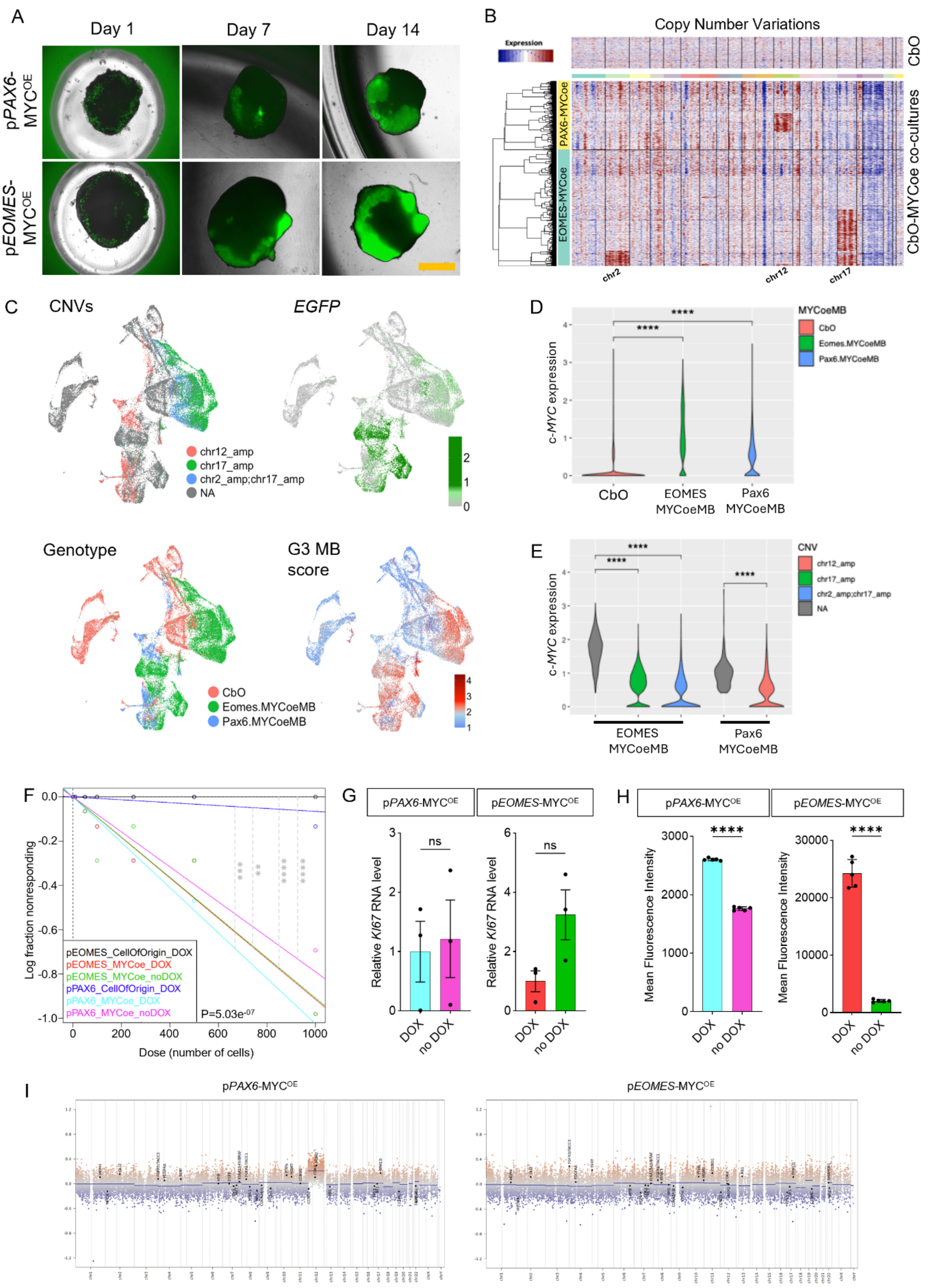
A
Day 1
Day 7
Day 14
pPAX6-MYCOE
pEOMES-MYCOE
B
Copy Number Variations
Expression
CbO
CbO-MYCoe co-cultures
PAX6-MYCoe
EOMES-MYCoe
chr2
chr12
chr17
C
CNVs
EGFP
chr12_amp
chr17_amp
chr2_amp;chr17_amp
NA
Genotype
G3 MB score
CbO
Eomes.MYCoeMB
Pax6.MYCoeMB
D
c-MYC expression
MYCoeMB
CbO
EOMES MYCoeMB
Pax6 MYCoeMB
E
CNV
F
Log fraction nonresponding
Dose (number of cells)
pEOMES_CellOfOrigin_DOX
pEOMES_MYCoe_DOX
pEOMES_MYCoe_noDOX
pPAX6_CellOfOrigin_DOX
pPAX6_MYCoe_DOX
pPAX6_MYCoe_noDOX
P=5.03e-07
G
pPAX6-MYCOE
pEOMES-MYCOE
Relative Ki67 RNA level
ns
DOX
no DOX
H
Mean Fluorescence Intensity
****
I
pPAX6-MYCOE
pEOMES-MYCOE

Supplementary Figure 5. MYC^{OE} cells acquire additional mutations when co-cultured and retain tumour phenotypes upon DOX withdrawal.

(A) Representative images of FAC-sorted GFP-tagged D103 p*PAX6*-MYC^{OE} or p*EOMES*-MYC^{OE} cells after 1, 7 or 14 days of co-culture with naïve CbO. Brightfield and GFP overlaid. Scale bar = 1 mm. **(B)** Hierarchical clustering of inferred copy number scRNA-Seq profiles separates co-cultured p*PAX6*-MYC^{OE} or p*EOMES*-MYC^{OE} cells from CbO cells. **(C)** UMAP plots of scRNA-Seq data from GFP-tagged D103 p*PAX6*-MYC^{OE} and p*EOMES*-MYC^{OE} cells after 14 days of co-culture with naïve CbO coloured by: copy number variants detected by inferCNV (top left; B); Alra-imputed *EGFP* (top right); Assigned genotype (bottom right); score for a Group 3 MB gene signature (Vladoiu et al. 2019). Pooled samples of n = 3 CbO/co-cultures per condition. **(D)** Violin plot showing alra-imputed c-*MYC* expression in naïve CbO cells vs co-cultured p*EOMES*-MYC^{OE} and p*PAX6*-MYC^{OE} . Wilcoxon test p-values are reported ****p<0.0001. **(E)** Violin plot showing alra-imputed c-*MYC* expression within co-cultured p*EOMES*-MYC^{OE} and p*PAX6*-MYC^{OE} only, separated by copy number variants detected. Wilcoxon test p-values are reported ****p<0.0001. **(F)** Extreme limiting dilution analysis of p*PAX6*/p*EOMES* FACS-sorted GFP+ MYC^{OE} or BFP+ cells-of-origin with and without doxycycline (DOX). Chi-square pairwise test ** p<0.01, ***p<0.001, ****p<0.0001. **(G)** qPCR analysis showing expression of *KI67* in MYC^{OE} cells with DOX or following 14 days of DOX withdrawal. CT values normalised to *ACTIN B* housekeeping (dCT) and expressed as 2^(-dCT) relative to DOX, graphs report mean ± SEM. **(H)** Mean fluorescence intensity of MYC^{OE} cells co-cultured with CbO61 for 14 days with or without DOX. Welch's unpaired t-test ****p<0.0001, graphs report mean ± SEM. **(I)** Genomic CNV profile for p*PAX6*-MYC^{OE} (left) and p*EOMES*-MYC^{OE} (right) from DNA samples of tumour xenografts (Capper et al. 2018).

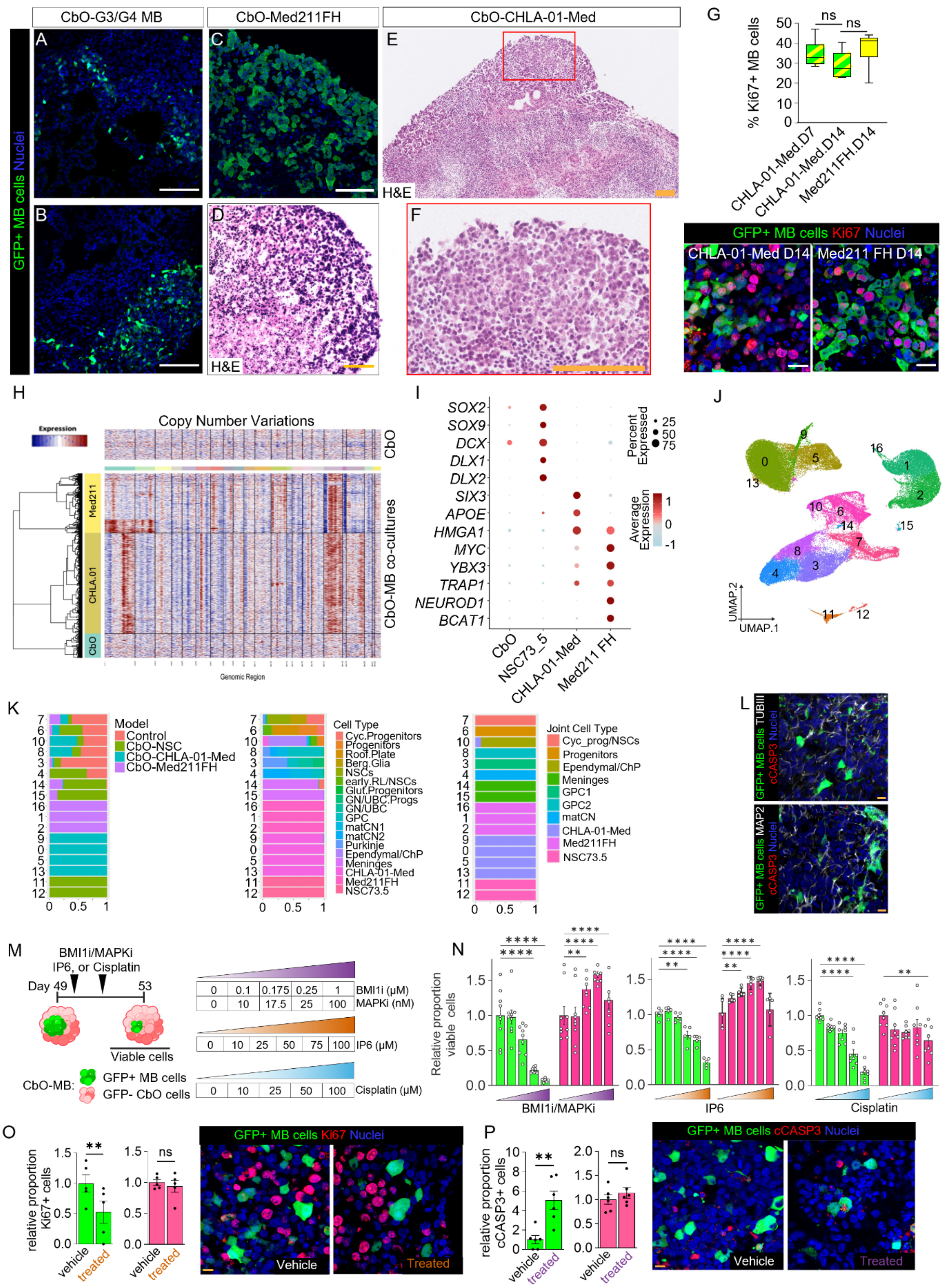
CbO-G3/G4 MB
CbO-Med211FH
CbO-CHLA-01-Med
GFP+ MB cells Nuclei
A
B
C
D
E
F
G
H&E
% Ki67+ MB cells
ns
ns
CHLA-01-Med.D7
CHLA-01-Med.D14
Med211FH.D14
GFP+ MB cells Ki67 Nuclei
CHLA-01-Med D14
Med211 FH D14
H
Copy Number Variations
Expression
CbO
CbO-MB co-cultures
Med211
CHLA.01
Genomic Region
I
SOX2
SOX9
DCX
DLX1
DLX2
SIX3
APOE
HMGA1
MYC
YBX3
TRAP1
NEUROD1
BCAT1
CbO
NSC73_5
CHLA-01-Med
Med211 FH
Percent Expressed
Average Expression
J
UMAP.1
UMAP.2
K
Model
Control
CbO-NSC
CbO-CHLA-01-Med
CbO-Med211FH
Cell Type
Cyc.Progenitors
Progenitors
Roof.Plate
Berg.Glia
NSCs
early.RL/NSCs
Glut.Progenitors
GN/UBC.Progs
GN/UBC
GPC
matCN1
matCN2
Purkinje
Ependymal/ChP
Meninges
CHLA-01-Med
Med211FH
NSC73.5
Joint Cell Type
Cyc_prog/NSCs
Progenitors
Ependymal/ChP
Meninges
GPC1
GPC2
matCN
CHLA-01-Med
Med211FH
NSC73.5
L
GFP+ MB cells cCASP3 Nuclei TUBIII
GFP+ MB cells cCASP3 Nuclei MAP2
M
BMI1i/MAPKi
IP6, or Cisplatin
Day 49
53
Viable cells
CbO-MB:
GFP+ MB cells
GFP- CbO cells
BMI1i (µM)
MAPKi (nM)
IP6 (µM)
Cisplatin (µM)
N
Relative proportion viable cells
BMI1i/MAPKi
IP6
Cisplatin
O
relative proportion Ki67+ cells
vehicle
treated
GFP+ MB cells Ki67 Nuclei
Vehicle
Treated
P
relative proportion cCASP3+ cells
GFP+ MB cells cCASP3 Nuclei

Supplementary Figure 6. MB cells display the morphology of human MB upon co-culture with CbO and retain their genetic features.
(A-C) Representative immunofluorescence staining of GFP+ ICb1299 G3/4 (A), CHLA-01R-Med (B) or Med211FH (C) MB cells upon fourteen days of co-culture with CbO_61, scale bars = 100 µm. **(D-F)** H&E staining of CbO-Med211FH (D), CbO-CHLA-01-Med (E) or CbO-Med211FH (F) co-cultures upon 14 days of co-culture, scale bar = 100 µm. **(G)** Box and whisker plot showing percentage of MB cells (GFP+) expressing the proliferative marker *Ki67* upon 7 and 14 days of co-culture in CHLA-01-Med and Med211FH cells. Graphs report mean ± SEM, unpaired t-test, n = 6 independent CbO-MB analysed, ns = not significant (upper). Representative immunofluorescence staining of GFP+ and KI67+ MB cells upon 14 days of co-culture with CbO, scale bars = 100 µm (lower). **(H)** Hierarchical clustering of inferred copy number profiles separates co-cultured CHLA-01-Med (suede) and CbO-Med211-FH (yellow) from CbO (cyan) cells. **(I)** Dot plot showing key marker genes that distinguish co-culture NSC73_5, CHLA-01-Med and CbO-Med211-FH cell populations from CbO. **(J)** UMAP plot of scRNA-Seq data from D49 control CbO_61, CbO-Med211-FH, CbO-CHLA-01-Med and control CbO-NSC73_5 co-cultures, coloured by Louvain clusters. Pooled samples of n = 3 CbO/co-cultures were submitted per condition. **(K)** Bar plot showing proportion of each cluster made up of cells from CbO_61 control, CbO-Med211-FH, CbO-CHLA-01-Med or CbO-NSC73_5 co-culture samples (left). Bar plot showing proportion of each cluster made up of cell labels computationally transferred from D35/49 controls (middle). Bar plot showing proportion of each cluster made up of newly assigned joint cell type labels (right). **(L)** Immunohistochemistry images of cerebellar organoids co-cultured with NSC73.5 cells for 14 days. Upper panel: GFP (green), cCASP3 (red) and TubIII (white); Lower panel: GFP (green), cCASP3 (red) and MAP2 (white). Scale bars = 10 µm. **(M)** Schematic of CbO-CHLA-01-Med treatment with tables reporting the concentrations of PTC209 (BMI1i), PD329501 (MAPKi), IP6 and cisplatin used. **(N)** Viability assays of GFP+ MB cells (green) and GFP- CbO cells (pink) in CbO-MB upon 4 days of treatment with increasing concentrations of BMI1 and MAPK inhibitors (left), IP6 (middle) or cisplatin (right) as shown in Figure 4G-I. All graphs report mean ± SEM, number of independent CbO analyzed is reported in each graph, one-way ANOVA *p<0.05, ***p<0.001, ****p<0.0001, ns = not significant. **(O)** Quantification (left) and representative images (right) of vehicle and treated (1 µM BMI1i, 100 nM MAPKi) CbO-CHLA-01 co-cultures from for GFP and cCASP3. Graphs report mean ± SEM, paired t-test **p<0.01; ns = not significant. n = 6 regions of interest quantified. Scale bars = 10 µm. **(P)** Quantification (left) and representative images (right) of vehicle and treated (100 µM IP6) CbO-CHLA-01-Med co-cultures stained for GFP and Ki67. Graphs report mean ± SEM, paired t-test **p<0.01; ns = not significant. n = 5 regions of interest quantified. Scale bars = 10 µm.

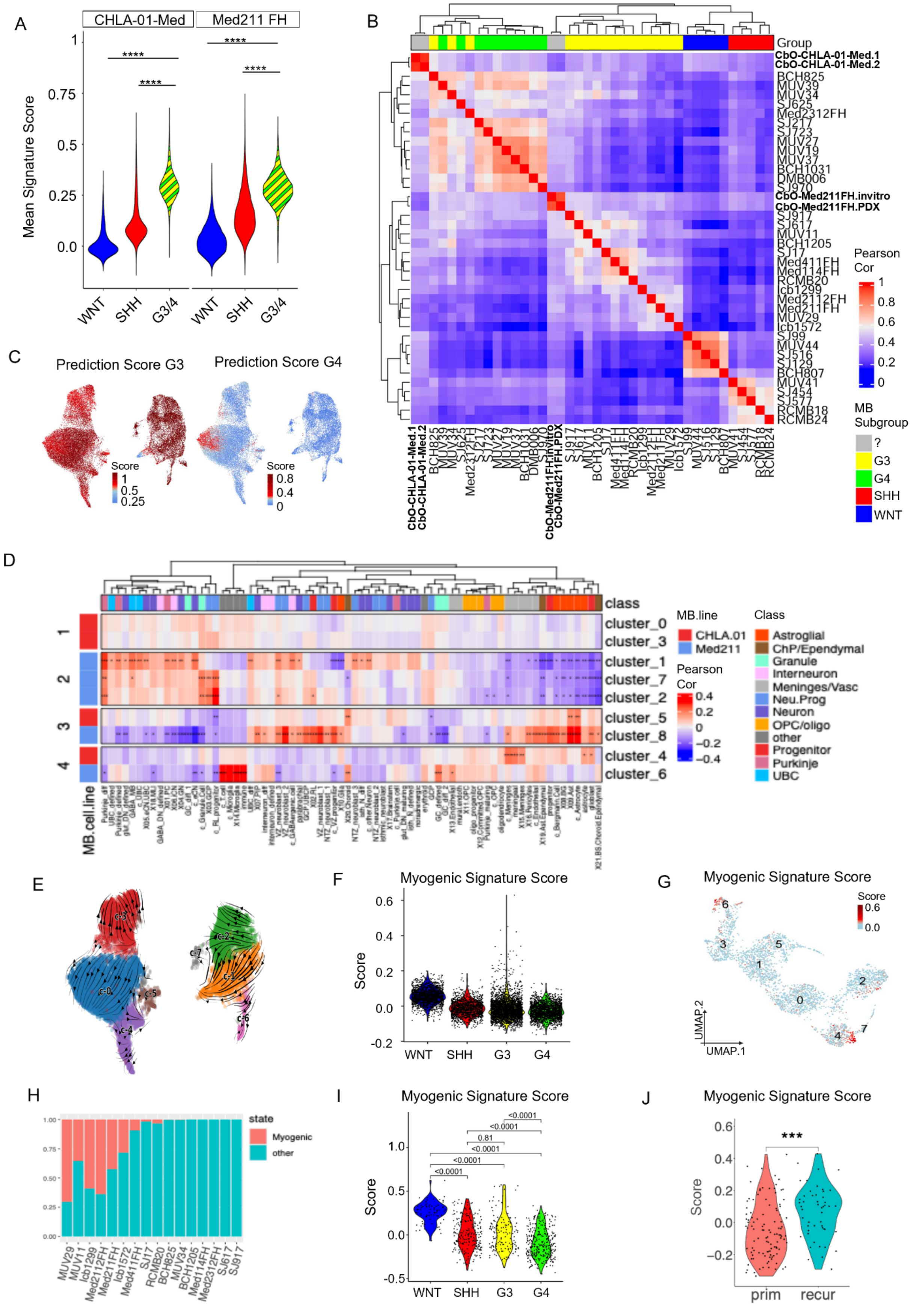

286
287

Supplementary Figure 7. Co-cultured MB cells retain the subgroup identity and transcriptional features of MB tumours.

(A) Violin plot showing signature scores for WNT, SHH or G3/G4 MB subgroups (Hovestadt et al. 2019) in CbO-MB. One-way ANOVA ****p<0.0001. **(B)** Heatmap showing correlation scores between expression of pseudo-bulk RNA-Seq of MB fractions only from CbO-Med211-FH and CbO-CHLA-01-Med and reference MB samples (Hovestadt et al. 2019) . Clustering based on expression profile has been used to assess molecular subgroup of CbO-MB models. **(C)** UMAP plot of scRNA-Seq data from MB cells only from D49 CbO-Med211-FH and CbO-CHLA-01-Med CbO-MB co-cultures, coloured by label transfer scores for Group 3 (left), Group 4 (right). **(D)** Heatmap showing Pearson correlation of co-cultured Med211-FH and CHLA-01-Med scRNA-Seq clusters to reference cell type from three independent scRNA-seq datasets of the human developing foetal cerebellum (Aldinger et al. 2021; Zhong et al. 2023; Sepp et al. 2024). Pearson correlation *p<0.05, **p<0.01, ***p<0.001. **(E)** UMAP plot of D49 CbO-Med211-FH and CbO-CHLA-01-Med CbO-MB co-cultures, RNA velocity streams shown, calculated in velocyto and scVelo (La Manno et al. 2018; Bergen et al. 2020). **(F)** Violin plot showing signature scores for the myogenic signature in different MB subgroups of a reference cohort of patient MB scRNA-Seq data (Hovestadt et al. 2019). **(G)** UMAP plot of Group 3 MB reference samples in H, coloured by myogenic signature score, and labelled by their Louvain cluster. **(H)** Bar plot showing the relative proportion of myogenic (clusters 4 and 6) vs non-myogenic cells in Group 3 MB reference samples in B. **(I)** Violin plot showing signature scores for the myogenic signature in different MB subgroups of a reference cohort of patient MB RNA-seq data (Cavalli et al. 2017). Wilcoxon test p-values are reported. **(J)** Violin plot showing signature scores for the myogenic signature in primary vs recurrent MB RNA-seq data (Hovestadt et al. 2019; Okonechnikov et al. 2023; Okonechnikov et al. 2025). Wilcoxon test ***p<0.001.

Supplementary Methods

Cerebellar Organoid Culture

EPSCs at 80% confluency were detached using a gentle cell dissociation reagent (StemCell) and plated into 96-well ultra-low attachment V-bottomed plates (PHCBI) in mTEsR plus basal media (StemCell) supplemented with 10 µM Y-27632 Dihydrochloride (PeproTech, #1293823) at 6,000 cells per well. On Day 1, media was replaced with mTEsR plus basal media. On Day 2, media was replaced with growth-factor free chemically defined media (gfCDM) containing: IMDM (Life Technologies)/Ham’s F-12 (Life Technologies) 1:1, 1% v/v chemically defined lipid concentrate (Life Technologies), 450 µM monothioglycerol (Sigma), 15 µg/ml apo-transferrin (Sigma), 5 mg/ml crystallization-purified BSA (Sigma), 50 U/ml penicillin/50 µg/ml streptomycin and 7 µg/ml insulin (Sigma). On Day 2-7, 50 ng/ml human recombinant FGF2 (PeproTech, #100-18B) and 10 µM SB431542 inhibitor (Life Sciences, #S4317) were added to gfCDM. On Day 7, organoids were transferred to 6-well ultra-low attachment plates (Corning) on an orbital shaker (90 rpm, Thermo Scientific, #88881102) in a humidified incubator at 37°C with 5% CO2 where they were maintained for the remainder of the protocol. For Day 7-14, 33.3 ng/ml human recombinant FGF2 and 6.67 µM SB431542 inhibitor were added to gfCDM, then 100 ng/ml human recombinant FGF19 (PeproTech, #100-32) was added to gfCDM for Day 14-21 of culture. From Day 21, organoids were cultured in complete neurobasal media containing: Neurobasal medium (Life Technologies), GlutaMax I (Life Technologies), N2 supplement (Life Technologies), and 50 U/ml penicillin/50 µg/ml streptomycin. 300 ng/ml human recombinant SDF1 (PeproTech, #300-28A) was added to complete neurobasal media from Day 28-35. Organoid size was monitored over the first 35 days of culture. The average diameter from three measurements per organoid was taken using ImageJ software. Relative growth was calculated as a fold change from the average diameters at each timepoint shown.

Human Cerebellar FFPE Samples Processing

12 human cases from Great Ormond Street Hospital (GOSH) at pre-natal developmental timepoints (19-37 post-conception weeks) were selected as having normal cerebellar histology and consent for research. Areas of formalin-fixed paraffin-embedded (FFPE) tissue containing cerebellar material were identified and manually

macro-dissected according to the marking shown in Figure S1D. Following dissection, the tissue was kept at -20°C until downstream processing. In addition, 11 samples of fresh frozen cerebellar tissue from earlier developmental timepoints (Carnegie stage 22-17 post-conception weeks) were obtained from the Human Developmental Biology Resource (HDBR).

Extreme Limiting Dilution Assays (ELDA) and Doxycycline Withdrawal

For ELDAs, GFP-sorted or BFP-sorted MYC^{OE} cells or MB cells-of-origin were plated into 96 well ultra-low attachment round-bottom plates at densities of 1, 10, 100, 250, 500 and 1000 cells/well with at least 8 replicates per condition. Cells were cultured in complete neurobasal CbO media with or without doxycycline (2 µg/mL) and monitored for sphere-formation for three weeks, with media changed weekly. ELDA data were analysed using the http://bioinf.wehi.edu.au/software/elda/ software (Hu and Smyth 2009). For doxycycline withdrawal GFP assays, >D103 CbO-MYC^{OE} co-cultures were established for 1 week then doxycycline withdrawn from media and culture continued for 2 weeks. After which, CbO-MYC^{OE} were dissociated and taken for flow cytometry as described in CbO-MB drug assays methods, with final analysis and MFI calculated in FlowJo v10.6.2.

Table 1 Primers used for RT-qPCR reactions

Gene	Forward Primer (5'-3')	Reverse Primer (5'-3')
ACTIN B	GCGAGAAGATGACCCAGATC	CCAGTGGTACGGCCAGAGG
GAPDH	CAATGACCCCTTCATTGACC	GACAAGCTTCCCGTTCTCAG
NESTIN	GAAACAGCCATAGAGGGCAAA	TGGTTTTCCAGAGTCTTCAGTGA
SOX1	TGCTTGTTCTGTTAACTCAC	AAAGAACCTCAGAGAGAGTC
EN2	CCGGCGTGGGTCTACTGTA	GGCCGCTTGTCCTCTTTGTT
FOXP2	ACATACATTCAATCCACGTC	CAGTTCTCATTCCAGATCTTC
OTX2	AGAGGACGACGTTCACTCG	TCGGGCAAGTTGATTTTCAGT
KIRREL2	ACAACTCTCCTTATGGTCATC	ATTCGCATCAGGTTCTTTTG
OLIG2	GACAAGCTAGGAGGCAGTGG	CGGCTCTGTCATTTGCTTCTTG
SKOR2	CCAGGTGTTAAAAGGAAACACA	GCTCCCTTTTCATCTGATCCT
PAX6	AGAGAATACCAACTCCATCAG	GATAATGGGTTCTCTCAAACTC
ATOH1	TGTTATCCCGTCGTTCAACAAC	TGGGCGTTTGTAGCAGCTC
BARHL1	GAGCGGCAGAAGTACCTGAG	GTAGAAATAAGGCGACGGGAAC
EOMES	CACATTGTAGTGGGCAGTGG	CGCCACCAAACTGAGATGAT
MYC	AGTGGAAAACCAGCAGCCTC	TTCTCCTCCTCGTCGCAGTA
GATA6	GCCAACTGTCACACCACAAC	TCATAGCAAGTGGTCTGGGC
NANOG	AGAAAAACAACTGGCCGAAGAAT	GTTGAATTGTTCCAGGTCTGGTT
MKI67	GACAGAGGTTCCTAAGAGAG	AACAATCAGATTTGCTTCCG

Table 2 Primary antibodies and dilutions used for immunohistochemistry

**Primary Antibody**	**Dilution**
SOX2 (abcam, ab97595)	1:500
OLIG2 (Santa Cruz, sc-19967)	1:200
CALB (Swant, CB300)	1:200
NeuN (Chemicon, MAB377)	1:500
EOMES (Chemicon, AB15894)	1:200
KI67 (abcam, ab15580)	1:500
GFP (Chemicon, AB16901)	1:400
cCASP3 (Cell Signalling, 9664)	1:500
MAP2 (Sigma, M4403)	1:200
TubIII (abcam, ab7751)	1:200
Myogenin (abcam, ab1835)	1:100
Synaptophysin (DAKO, M731529-2)	1:500
Human Vimentin (Roche, 790-2917)	Pre-diluted
TGFB3 (abcam, ab15537)	1:200
TGFBR3 (Santa Cruz, sc-74511)	1:200

scRNA-Seq: Methodological details of downstream analyses

UMAP calculations and cell type annotations

For analysis of CbO samples we implemented stringent quality filtration, only cells with fewer than $5x10^4$ total reads, lower than 10% mitochondrial reads, more than 750 and less than 6,500 detected features were retained for analysis. Expression values were library size corrected to 10,000 reads per cell and log1p transformed, with Principal component analysis (PCA) performed on the scaled data for the top 2,000 variable genes. Batch correction between cell lines was performed on principal components using Harmony (Korsunsky et al. 2019). Uniform Manifold Approximation Projection (UMAP) embeddings, Nearest Neighbours and cell clusters were then calculated in harmony-corrected PCA space using 35 dimensions, and cells were clustered using FindClusters() with a resolution of 0.5 and used FindSubCluster(resolution = 0.1, algorithm = 1) to separate cluster 7 into two distinct subclusters 7_0 and 7_1. Cluster marker genes were calculated using a Seurat's in-bult Wilcoxon Rank Sum test (logfc.threshold = 0.25, min.pct = 0.1, only.pos = T), and differential expression analysis across experimental conditions was performed using MAST (Finak et al. 2015). Initial clustering identified two clusters, 13 and 14, which represented hypoxic/glycolytic cells, common to all human brain organoids(Bhaduri et al. 2020). As has been done previously (Kanton et al. 2019), these cells were excluded from analyses, and a new UMAP was calculated.

For analysis of engineered CbO and CbO co-cultures containing c-MYC-GFP–expressing CbO cells, either CHLA-01-Med or Med211FH medulloblastoma (MB) cells, or control NSC37.5 cells, we changed the detected feature threshold to greater than 500 and less than 7,500, with no batch correction necessary. For engineered CbO UMAPs and Nearest neighbours were calculated using 30 PCA dimensions, and FindClusters() was run with a resolution of 0.5. For CbO co-cultures with c-MYC-GFP–expressing CbO cells, cell cycle signature scores were regressed during data scaling(vars.to.regress = c("S.Score","G2M.Score"), and UMAP embeddings were calculated on PCA 35 dimensions. InferCNV was run using D49 CbO cells as a reference, the "subclusters" analysis mode, and setting the following parameters: cutoff=0.1, leiden_resolution = $2.5x10^{-5}$. Cells with either a detectable CNV or alra-imputed (Linderman et al. 2022) EGFP expression > 0.25 were assigned as c-MYC-

GFP–expressing CbO cells. For CbO co-cultures with CHLA-01-Med or Med211FH medulloblastoma (MB) cells, or control NSC37.5 cells, we used 15 PCA dimensions and a resolution of 0.35. Malignant CHLA-01-Med and Med211FH MB cells were distinguished from co-cultured CbO cells based on Leiden clustering and inferred copy number variants, with co-cultured NSC73.5 identified based on clustering and marker gene expression alone. InferCNV was run on CbO-CHLA-01-Med and CbO-Med211FH samples using D49 CbO cells as a reference, the "subclusters" analysis mode, and setting the following parameters: cutoff=0.1, k_obs_groups = 3, leiden_resolution = $1x10^{-5}$. For annotating engineered CbO and organoid cells from CbO-MB co-cultures we used the processed R object from the D35-49 CbO (Figure 2A) as a reference, and the default settings of Seurat FindTransferAnchors(), TransferData() and AddMetaData() functions. We then annotated new cluster names based on their composition of transferred cell type labels.

Finally, for the analysis of MB cells alone, CHLA-01-Med and Med211FH cells identified by InferCNV were subset and reanalysed using the standard Seurat pipeline; UMAPs and nearest neighbours were calculated using 20 PCA dimensions and FindClusters() was run with resolution=0.225.

scRNA-Seq Analysis: Comparison to reference datasets

To aid with D35/49 CbO cell type annotations, we compared cluster marker signatures against those from three foetal cerebellum datasets (Aldinger et al. 2021; Zhong et al. 2023; Sepp et al. 2024). In each case we obtained published count matrices, UMAP-embeddings and author-defined cluster metadata, and used them to perform a Wilcoxon Rank Sum test with Seurat's FindAllMarkers() command (logfc.threshold = 0.25, min.pct = 0.1, only.pos = T) to obtain the list of marker genes for their cell types. Then, for each dataset and marker gene, cluster specificity scores were computed (mean normalised counts per cluster/total mean normalised counts) – with overlapping gene signature specificity scores compared across studies by Pearson correlation. We performed the same analysis on CHLA-01-Med and Med211FH cells to determine each MB cluster's closest analogues from developing foetal cerebellum samples.

To compare CHLA-01-Med and Med211FH MB cells in co-cultures to patient tumours, we generated pseudo-bulk transcriptomic data from aggregated single-cell counts

from our co-cultured MB samples, and 36 patients in Hovestadt et al (Hovestadt et al. 2019). Combat batch correction was performed between log(cpm+1) transformed data, and Pearson correlation between samples was calculated across the top 2,500 most variable genes (ranked by standard deviation). Pearson correlation across the same gene list was also used to query the relative similarity between patient tumours and CbO co-cultured CHLA-01-Med cells profile here, and CHLA-01-Med cells we previously cultured *in vitro* and profiled by RNAseq(Badodi et al. 2021). For Med211FH MB cells, we compared CbO co-cultured to RNAseq data from an *in vitro* sample drawn from the literature (Visvanathan et al. 2024) and a second *in vitro* sample profiled as part of this study.

scRNA-Seq Analysis: Reference gene set/signature scoring

For reference signature scoring, average gene module expression was calculated for every single cell, subtracted by the aggregated expression of a random control set of features selected from the same average expression bins as the query genes (Venteicher et al. 2017) using Seurat's AddModuleScore() function. G/S and G2M gene modules are included in the Seurat v5.0.1 package; G3 RL.PRC and G4 RL.UBC Medulloblastoma cell of origin signatures were taken from Smith et al (Smith et al. 2022); Group 3 (G3) and 4 (G4) MB tumour signatures were taken from Vladoiu et al (Vladoiu et al. 2019); WNT, SHH and G3/4 MB cell state signatures were taken from Hovestad et al (Hovestadt et al. 2019). For gene set enrichment analysis, gene sets were obtained from MolSigDB (Liberzon et al. 2015) and computations were performed using the R package fGSEA v4.4 (Korotkevich et al. 2016). Gene ontology enrichment analysis was performed on upregulated (p.adj<0.05, and avg_log2FC>2) genes in the MYC^{OE} cluster of engineered CbOs using the topGO v2.52.0 and org.Hs.eg.db v3.17.0 R packages.

Processed cell count and metadata from patient-derived MB samples were accessed from Hovestadt et al(Hovestadt et al. 2019) and reanalysed in Seurat using default settings. The resulting R object was then used as a reference, with the default settings of Seurat FindTransferAnchors(), TransferData() and AddMetaData() functions to assign G3 and G4 prediction scores to the co-cultured CHLA-01-Med and Med211FH MB cells. To generate a Myogenic MB state signature, we took the intersect of significant MB marker genes with avg_log2FC>1 from cluster 4 (CHLA-01-Med) and

cluster 6 (Med211FH). The processed reference R object from Hovestadt et al (Hovestadt et al. 2019) was then scored for the Myogenic MB state signature as detailed above using the AddModuleScore() function. Next, the Hovestadt et al(Hovestadt et al. 2019) data was subset to G3 samples, before Harmony(Korsunsky et al. 2019) batch correction between samples was performed. New UMAP embeddings, Nearest Neighbours were then calculated in harmony-corrected PCA space using 15 dimensions, and clusters were identified using a resolution of 0.5. We also analysed a large reference dataset of 763 bulk MB RNAseq samples (Cavalli et al (Cavalli et al. 2017)) for expression of the Myogenic MB state signature using the GSVA 1.48.3 R package (Hanzelmann et al. 2013). Survival analysis was performed on patients with complete data (i.e. that had died) using the survival_3.8-3 and survminer_0.5.0 R packages and splitting patients into the Myogenic Signature High or Low groups based on the median signature score. For myogenic signature scoring in recurrent MB, data was drawn from three studies containing both primary and recurrent samples (Hovestad et al. 2017; Okonechnikov et al., 2023; Okonechnikov et al., 2025), scRNAseq data was pseudobulked and socred alongside RNAseq samples using the GSVA 1.48.3 R package (Hanzelmann et al. 2013).

Cell lineage trajectory analysis

For RNAvelocity analysis, velocyto (La Manno et al. 2018) was used to calculate spliced and unspliced transcriptomes, before subsequent analysis using scVelo (Bergen et al. 2020), on Seurat-defined cell types and UMAP embeddings, using default parameters. For single-cell lineage trajectory analysis, we imported the processed Seurat object into the Monocle3 R package. The 'learn_graph' function was applied to infer single cell trajectories setting close_loop = TRUE, minimal_branch_len to 10 and euclidean_distance_ratio to 1.5. The 'order_cells' function was then used to set Progenitors and Cyc_Progenitors as the root of states of pseudotime. Next we subset data to cell types involved in the G3 trajectory (early_RL/NSCs, glut_progenitors and matCN2) or G4 trajectory (NSCs, GN/UBC_progs and GN/UBC) (Cao et al. 2019). For each subset, the 'learn_graph' function was applied again to infer single cell trajectories setting minimal_branch_len to 10 and the 'order_cells' function was used to select early_RL/NSCs and NSCs as the root states of pseudotime. Differential gene expression was calculated across single-cell trajectories using Moran's I test. For each trajectory, the top 2500 most differentially expressed

genes over pseudotime were selected, and smoothened, alra-imputed (Linderman et al. 2022) expression over pseudotime was plotted.

Receptor Ligand Modelling

For receptor-ligand analysis, cell type meta data and count data for the top 15000 most highly variable genes in the CbO-MB dataset was input into CellphoneDB v5 (Efremova et al. 2020) and run using the statistical_analysis mode.

DNA methylation: Methodological details of downstream analyses

Cerebellar Organoid developmental staging

To stage the development of CbO compared with human cerebellar samples, an 'ageing signature' approach was applied, with the top 1% of probes whose beta values best correlated to age in human cerebellar samples selected for subsequent correlation with CbO probe betas. Combat batch correction was first performed between CbO and human samples before top 1% probe selection and correlation to CbO. To corroborate this approach, CbO methylation data adapted to EPIC v1 array probe annotation were also analysed with the foetal brain clock (FBC), an R package specifically trained on foetal brain samples during development to estimate their epigenetic age (Steg et al. 2021).

Differentially and variably methylated probes and regions analysis

Differentially methylated probes (DMPs) and regions (DMRs) were calculated pairwise for CbO samples between comparison groups using the R package DMRcate (Peters et al. 2015) with an FDR threshold of 0.01. Additional criteria for DMR identification were set such that DMRs must contain at least 6 CpG sites with <400 base pair separation. DMRs were mapped to gene promoters using the annotatr R package. Variable methylated probes (VMPs) and regions (VMRs) across CbO and human development timepoints were identified within the DMRcate package using the same thresholds as stated above for DMR analysis. Significant VMRs were then annotated to genes (VMGs), after which VMGs were clustered based on z-scores of average beta-values of the probes contained within them (k-means k = 5 set based on timepoint-dependent methylation programs) and taken for pathway analysis. For pathway analysis, gene lists were input into Gprofiler (Kolberg et al. 2023) and

pathways calculated based on a Benjamini-Hochberg FDR < 0.05. Pathway visualisation was performed using CytoScape (Shannon et al. 2003). The top 100 most significant (adjusted-p<0.05) GO:Biological Process and GO:Molecular Function pathways were plotted for human and CbO VMG clusters. For VMG comparison, average betas for human reference VMRs mapping to common VMGs were calculated, and Pearson correlation was performed between human and CbO samples.

DNA methylation: Comparison to reference datasets

For human rhombic lip (RL) and external granular layer (EGL) correlation, raw .idat files for micro-dissected RL and EGL samples were accessed from GSE207266 (Smith et al. 2022) and processed using the same raw data processing pipeline adapted for EPIC v1 array annotation before downstream analysis. DMPs were identified between each cerebellar compartment (n=7) as stated above with an FDR threshold of 0.01. DMPs were then subset to CbO average probe betas adapted to EPIC v1 array annotation and correlated to each cerebellar compartment. For comparison to previously published MB and organoid samples (Ballabio et al. 2020), MYC^{OE} xenograft probe names were adapted to EPIC v1 annotation and ComBat batch correction between studies performed before multidimensional scaling analysis taking the top 1000 most variable probes across all samples.

scRNA-Seq/DNA methylation integration analysis

Differentially expressed genes (DEGs) between CbO scRNA-Seq data with adjusted p-value < 0.05 and absolute log2FC > 0.25 were integrated with DMRs mapping to gene promoters from matched methylation samples with p-value < 0.05 and absolute average beta-value difference > 0.1. For pathway analysis, gene lists were input into Gprofiler (Kolberg et al. 2023) and pathways calculated based on a Benjamini-Hochberg FDR < 0.05.

References

Aldinger KA, Thomson Z, Phelps IG, Haldipur P, Deng M, Timms AE, Hirano M, Santpere G, Roco C, Rosenberg AB et al. 2021. Spatial and cell type transcriptional landscape of human cerebellar development. *Nat Neurosci* **24**: 1163-1175.

Badodi S, Pomella N, Zhang X, Rosser G, Whittingham J, Niklison-Chirou MV, Lim YM, Brandner S, Morrison G, Pollard SM et al. 2021. Inositol treatment inhibits medulloblastoma through suppression of epigenetic-driven metabolic adaptation. *Nat Commun* **12**: 2148.

Ballabio C, Anderle M, Gianesello M, Lago C, Miele E, Cardano M, Aiello G, Piazza S, Caron D, Gianno F et al. 2020. Modeling medulloblastoma in vivo and with human cerebellar organoids. *Nat Commun* **11**: 583.

Bergen V, Lange M, Peidli S, Wolf FA, Theis FJ. 2020. Generalizing RNA velocity to transient cell states through dynamical modeling. *Nature Biotechnology* **38**: 1408-1414.

Bhaduri A, Andrews MG, Mancia Leon W, Jung D, Shin D, Allen D, Jung D, Schmunk G, Haeussler M, Salma J et al. 2020. Cell stress in cortical organoids impairs molecular subtype specification. *Nature* **578**: 142-148.

Cao J, Spielmann M, Qiu X, Huang X, Ibrahim DM, Hill AJ, Zhang F, Mundlos S, Christiansen L, Steemers FJ et al. 2019. The single-cell transcriptional landscape of mammalian organogenesis. *Nature* **566**: 496-502.

Cavalli FMG, Remke M, Rampasek L, Peacock J, Shih DJH, Luu B, Garzia L, Torchia J, Nor C, Morrissy AS et al. 2017. Intertumoral Heterogeneity within Medulloblastoma Subgroups. *Cancer Cell* **31**: 737-754 e736.

Efremova M, Vento-Tormo M, Teichmann SA, Vento-Tormo R. 2020. CellPhoneDB: inferring cell-cell communication from combined expression of multi-subunit ligand-receptor complexes. *Nat Protoc* **15**: 1484-1506.

Finak G, McDavid A, Yajima M, Deng J, Gersuk V, Shalek AK, Slichter CK, Miller HW, McElrath MJ, Prlic M et al. 2015. MAST: a flexible statistical framework for assessing transcriptional changes and characterizing heterogeneity in single-cell RNA sequencing data. *Genome Biology* **16**: 278.

Hanzelmann S, Castelo R, Guinney J. 2013. GSVA: gene set variation analysis for microarray and RNA-seq data. *BMC Bioinformatics* **14**: 7.

Hovestadt V, Smith KS, Bihannic L, Filbin MG, Shaw ML, Baumgartner A, DeWitt JC, Groves A, Mayr L, Weisman HR et al. 2019. Resolving medulloblastoma cellular architecture by single-cell genomics. *Nature* **572**: 74-79.

Hu Y, Smyth GK. 2009. ELDA: extreme limiting dilution analysis for comparing depleted and enriched populations in stem cell and other assays. *J Immunol Methods* **347**: 70-78.

Kanton S, Boyle MJ, He Z, Santel M, Weigert A, Sanchis-Calleja F, Guijarro P, Sidow L, Fleck JS, Han D et al. 2019. Organoid single-cell genomic atlas uncovers human-specific features of brain development. *Nature* **574**: 418-422.

Kolberg L, Raudvere U, Kuzmin I, Adler P, Vilo J, Peterson H. 2023. g:Profiler-interoperable web service for functional enrichment analysis and gene identifier mapping (2023 update). *Nucleic Acids Res* **51**: W207-W212.

Korotkevich G, Sukhov V, Budin N, Shpak B, Artyomov MN, Sergushichev A. 2016. Fast gene set enrichment analysis. *bioRxiv*.

Korsunsky I, Millard N, Fan J, Slowikowski K, Zhang F, Wei K, Baglaenko Y, Brenner M, Loh PR, Raychaudhuri S. 2019. Fast, sensitive and accurate integration of single-cell data with Harmony. *Nat Methods* **16**: 1289-1296.

La Manno G, Soldatov R, Zeisel A, Braun E, Hochgerner H, Petukhov V, Lidschreiber K, Kastriti ME, Lönnerberg P, Furlan A et al. 2018. RNA velocity of single cells. *Nature* **560**: 494-498.

Liberzon A, Birger C, Thorvaldsdottir H, Ghandi M, Mesirov JP, Tamayo P. 2015. The Molecular Signatures Database (MSigDB) hallmark gene set collection. *Cell Syst* **1**: 417-425.

Linderman GC, Zhao J, Roulis M, Bielecki P, Flavell RA, Nadler B, Kluger Y. 2022. Zero-preserving imputation of single-cell RNA-seq data. *Nat Commun* **13**: 192.

Peters TJ, Buckley MJ, Statham AL, Pidsley R, Samaras K, Lord RV, Clark SJ, Molloy PL. 2015. De novo identification of differentially methylated regions in the human genome. *Epigenetics & Chromatin* **8**.

Sepp M, Leiss K, Murat F, Okonechnikov K, Joshi P, Leushkin E, Spanig L, Mbengue N, Schneider C, Schmidt J et al. 2024. Cellular development and evolution of the mammalian cerebellum. *Nature* **625**: 788-796.

Shannon P, Markiel A, Ozier O, Baliga NS, Wang JT, Ramage D, Amin N, Schwikowski B, Ideker T. 2003. Cytoscape: a software environment for integrated models of biomolecular interaction networks. *Genome Res* **13**: 2498-2504.

Smith KS, Bihannic L, Gudenas BL, Haldipur P, Tao R, Gao Q, Li Y, Aldinger KA, Iskusnykh IY, Chizhikov VV et al. 2022. Unified rhombic lip origins of group 3 and group 4 medulloblastoma. *Nature* **609**: 1012-1020.

Steg LC, Shireby GL, Imm J, Davies JP, Franklin A, Flynn R, Namboori SC, Bhinge A, Jeffries AR, Burrage J et al. 2021. Novel epigenetic clock for fetal brain development predicts prenatal age for cellular stem cell models and derived neurons. *Mol Brain* **14**: 98.

Venteicher AS, Tirosh I, Hebert C, Yizhak K, Neftel C, Filbin MG, Hovestadt V, Escalante LE, Shaw ML, Rodman C et al. 2017. Decoupling genetics, lineages, and microenvironment in IDH-mutant gliomas by single-cell RNA-seq. *Science* **355**: eaai8478.

Visvanathan A, Saulnier O, Chen C, Haldipur P, Orisme W, Delaidelli A, Shin S, Millman J, Bryant A, Abeysundara N et al. 2024. Early rhombic lip Protogenin(+ve) stem cells in a human-specific neurovascular niche initiate and maintain group 3 medulloblastoma. *Cell* **187**: 4733-4750 e4726.

Vladoiu MC, El-Hamamy I, Donovan LK, Farooq H, Holgado BL, Sundaravadanam Y, Ramaswamy V, Hendrikse LD, Kumar S, Mack SC et al. 2019. Childhood cerebellar tumours mirror conserved fetal transcriptional programs. *Nature* **572**: 67-73.

Zhong S, Wang M, Huang L, Chen Y, Ge Y, Zhang J, Shi Y, Dong H, Zhou X, Wang B et al. 2023. Single-cell epigenomics and spatiotemporal transcriptomics reveal human cerebellar development. *Nat Commun* **14**: 7613.
